# Supplementary material for: Structural basis for triacylglyceride extraction from mycobacterial inner membrane by MFS transporter Rv1410
Source: Nat Commun. 2023 Oct 13;14:6449. doi: 10.1038/s41467-023-42073-0 (PMC10576003; doi:10.1038/s41467-023-42073-0)
Supplement: Supplementary file 1 — Supplementary Information [file 41467_2023_42073_MOESM1_ESM.pdf]

Structural basis for triacylglyceride extraction from mycobacterial inner membrane by MFS transporter Rv1410

Supplementary Information

Sille Remm[1], Dario De Vecchis[2], Jendrik Schöppe[3,4], Cedric A.J. Hutter[1,5], Imre Gonda[1], Michael Hohl[1,6], Simon Newstead[7], Lars V. Schäfer[2,*], Markus A. Seeger[1,8,*]

[1]Institute of Medical Microbiology, University of Zürich, Zürich, Switzerland

[2]Center for Theoretical Chemistry, Ruhr University Bochum, Bochum, Germany

[3]Institute of Biochemistry, University of Zürich, Zürich, Switzerland

[4]Present address: Novo Nordisk, København, Denmark

[5]Present address: Linkster Therapeutics, Zürich, Switzerland

[6]Present address: Department of Infectious Disease, Imperial College London, London, United Kingdom

[7]Department of Biochemistry, University of Oxford, Oxford, United Kingdom

[8]National Center for Mycobacteria, Zurich, Switzerland

*corresponding authors: lars.schaefer@ruhr-uni-bochum.de, m.seeger@imm.uzh.ch

Table of Contents

Supplementary Figures 3

Supplementary Tables 24

Supplementary Note 1: Rationale for mutant design 33

- Unique ion lock mutations 33
- D22/D35 33
- β-hairpin truncation 33
- Truncation of linker helices 34
- Mutations in lateral clefts 34
- Mutations in central cavity 34
- Truncations of periplasmic helix extensions 35
- TM12 mutations 35

Supplementary Note 2: Description of MD simulations 36

- Lipid interactions in $MHAS2168^{OUT}$ and $MHAS2168^{IN}$ 36
- TAGs in $MHAS2168^{OUT}$ and $MHAS2168^{IN}$ central cavities 36
- TAG transfer from $MHAS2168^{OUT}$ to LprG 38

Supplementary References 40

Supplementary Source Data 41

Supplementary Figures

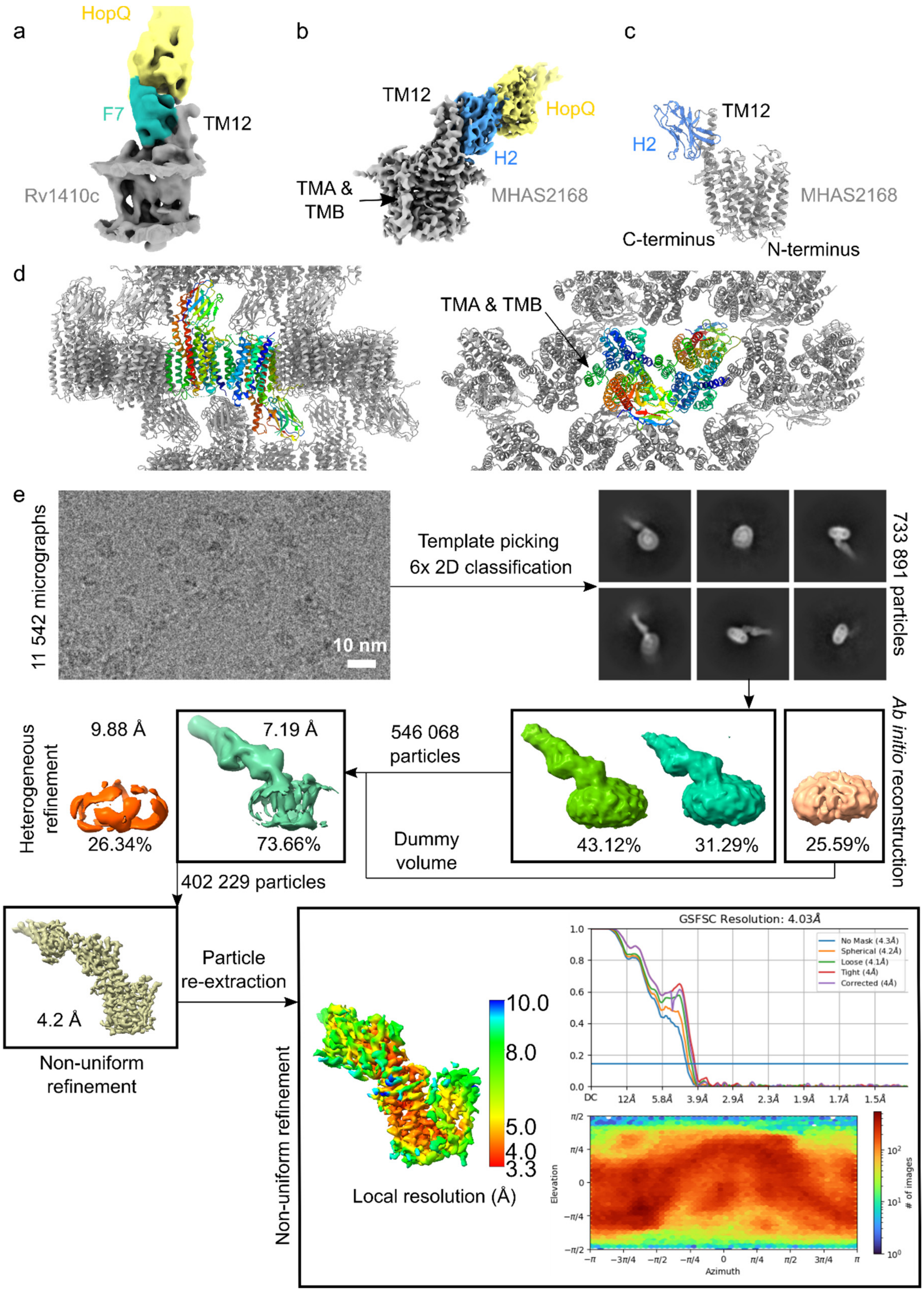

Supplementary Figure 1. Determination of Rv1410 and MHAS2168 structure. (a) 7.5 Å cryo-EM map of Rv1410-Mb_F7 complex. Rv1410 – gray; Nanobody F7 – sea green; Megabody HopQ domain – yellow. (b) 4.0 Å cryo-EM map of MHAS2168-Mb_H2 complex. MHAS2168 – gray; Nanobody H2 – blue; Megabody HopQ domain – yellow. TMA & TMB – linker helices A and B. (c) 2.7 Å crystal structure of MHAS2168-Nb_H2 complex. MHAS2168 – gray; Nanobody H2 – blue. (d) Crystal packing of MHAS2168-Nb_H2 complex lipidic cubic phase (LCP) crystals. The asymmetric unit comprises two transporter/nanobody complexes (rainbow color scheme). (e) Cryo-EM reconstruction of MHAS2168 in complex with Mb_H2. Representative cryo-EM image and 2D class averages of vitrified MHAS2168-Mb_H2 complex and image processing workflow. Final reconstructed map colored by local resolution as estimated in CryoSPARC. Fourier shell correlation (FSC) plot of non-uniform refinement with FSC threshold at 0.143 used for resolution estimation. Viewing direction distribution plot.

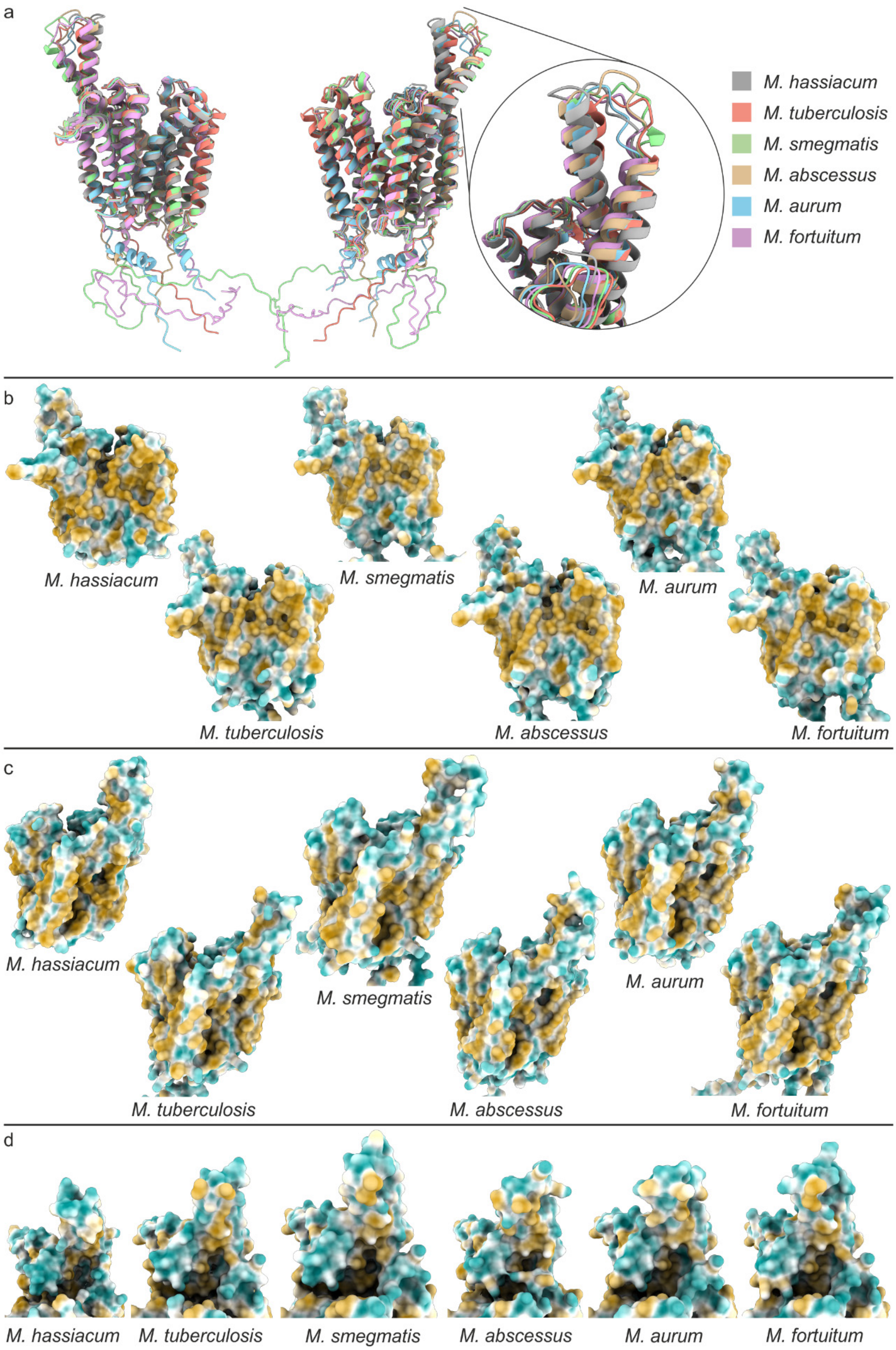
a
M. hassiacum
M. tuberculosis
M. smegmatis
M. abscessus
M. aurum
M. fortuitum
b
M. hassiacum
M. smegmatis
M. aurum
M. tuberculosis
M. abscessus
M. fortuitum
c
M. hassiacum
M. smegmatis
M. aurum
M. tuberculosis
M. abscessus
M. fortuitum
d
M. hassiacum
M. tuberculosis
M. smegmatis
M. abscessus
M. aurum
M. fortuitum

Supplementary Figure 2. Comparison of MHAS2168 crystal structure and its mycobacterial homologues' structure predictions. A subset of ColabFold structure predictions in outward-facing conformation that were used to analyze the common features of mycobacterial Rv1410/MHAS2168 homologues are shown together with the MHAS2168 crystal structure. (a) Structure predictions of homologues from *M. tuberculosis* (pale red), *M. smegmatis* (green), *M. abscessus* (beige), *M. aurum* (light blue), and *M. fortuitum* (pale purple) are superimposed on the crystal structure of MHAS2168 from *M. hassiacum* (gray). Inset: more detailed view of the TM11-TM12 periplasmic extensions shows differences in TM11 and loop length in different mycobacterial homologues while TM12 length remains the same. (b) Side views of hydrophobic surfaces of different mycobacterial homologues. The TM5-TM8^{OUT} lateral openings are narrow or (partially) obstructed. (c) Opposite side views of hydrophobic surfaces of different mycobacterial homologues. Linker helices TMA and TMB are blocking the TM2-TM11^{OUT} lateral opening. (d) Close-up view of the hydrophobic surfaces of the TM11-TM12 periplasmic extensions towards the central cavity. Hydrophobic patches are the common denominator of all periplasmic helix extensions. On panels (b) – (d), from left: homologues from *M. hassiacum*, *M. tuberculosis*, *M. smegmatis*, *M. abscessus*, *M. aurum*, *M. fortuitum*. On panels (b) – (d) hydrophobicity color scheme: hydrophobic – gold; hydrophilic – cyan.

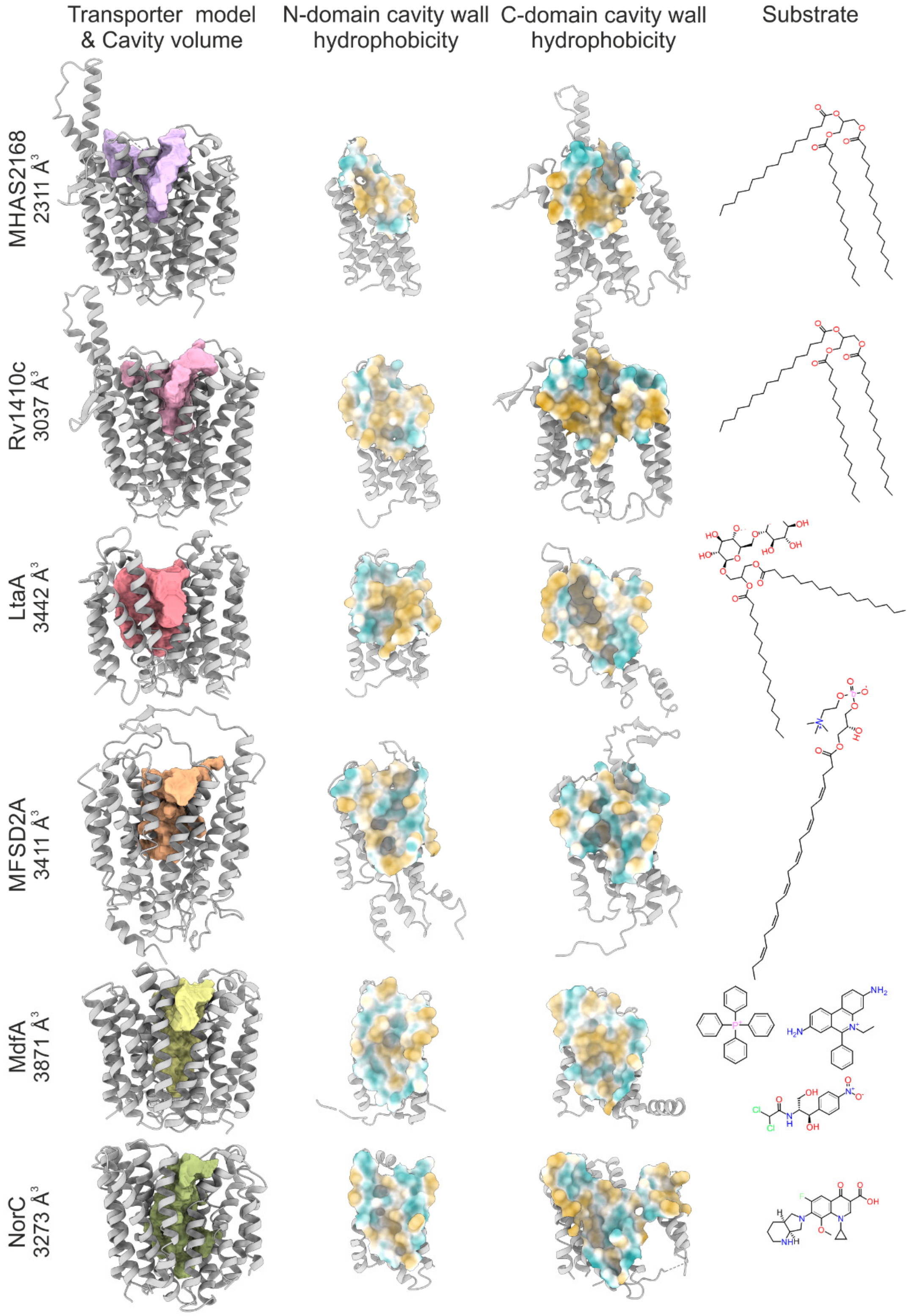
Transporter model & Cavity volume
N-domain cavity wall hydrophobicity
C-domain cavity wall hydrophobicity
Substrate
MHAS2168 2311 Å³
Rv1410c 3037 Å³
LtaA 3442 Å³
MFSD2A 3411 Å³
MdfA 3871 Å³
NorC 3273 Å³

Supplementary Figure 3. Comparison of central cavities from different MFS transporters known to transport lipids or drugs. The central cavity surface hydrophobicity/hydrophilicity reflects the polarity of the transporter's substrate(s). Left column: Side views of transporters (gray) with their central cavity volumes highlighted in color. Middle left column: Inside view of the hydrophobicity surfaces of the central cavity walls from N-domains. Middle right column: Inside view of the hydrophobicity surfaces of the central cavity walls from C-domains. Right column: Structures of substrates transported by the corresponding MFS transporters. From top to bottom: TAG exporter MHAS2168 crystal structure (this study, PDB ID: 8PNL) and TAG species tripalmitoylglycerol; TAG exporter Rv1410 ColabFold structure prediction (this study) and TAG species tripalmitoylglycerol; Lipoteichoic acid lipid anchor flippase LtaA crystal structure (PDB ID: 6S7V); Lysophosphatidylcholine-docosahexaenoic acid importer MFSD2A cryo-EM structure (PDB ID: 7N98); Multi-drug efflux pump MdfA crystal structure (PDB ID: 6GV1) and its substrates tetraphenylphosphonium, ethidium, and chloramphenicol; Quinolone efflux pump NorC crystal structure (7D5P) and its substrate moxifloxacin. Hydrophobicity color scheme: hydrophobic – gold; hydrophilic – cyan.

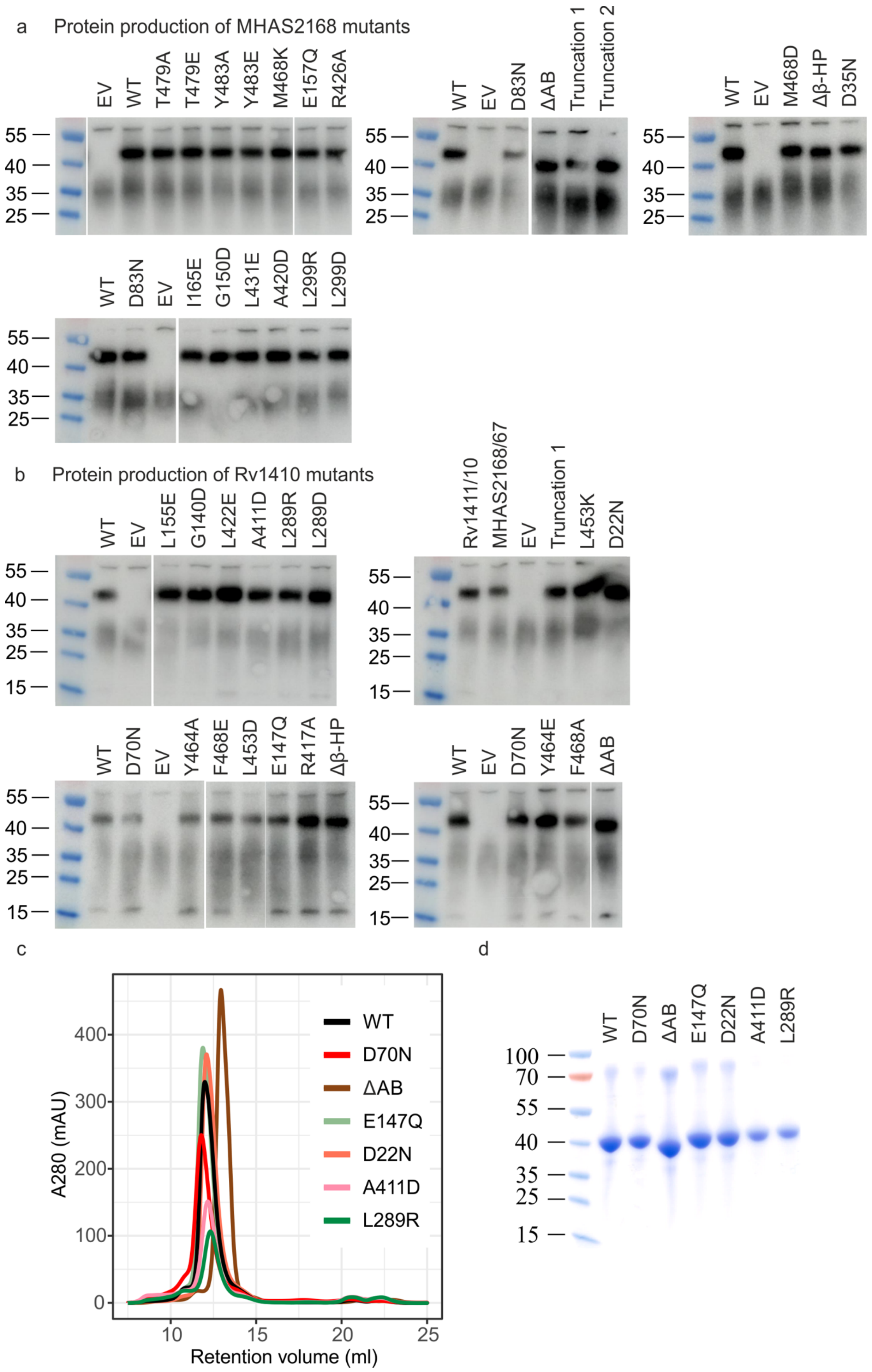
a
Protein production of MHAS2168 mutants
EV
WT
T479A
T479E
Y483A
Y483E
M468K
E157Q
R426A
D83N
ΔAB
Truncation 1
Truncation 2
M468D
Δβ-HP
D35N
I165E
G150D
L431E
A420D
L299R
L299D
55
40
35
25
b
Protein production of Rv1410 mutants
L155E
G140D
L422E
A411D
L289R
L289D
Rv1411/10
MHAS2168/67
L453K
D22N
D70N
Y464A
F468E
L453D
E147Q
R417A
Y464E
F468A
15
c
WT
D70N
ΔAB
E147Q
D22N
A411D
L289R
A280 (mAU)
Retention volume (ml)
0
100
200
300
400
10
20
d
100
70

Supplementary Figure 4. Validation of Rv1410 and MHAS2168 mutants. Western blot analysis of protein production levels of MHAS2168 and MHAS2168 mutants (a) and Rv1410 and Rv1410 mutants (b) show no aggregation or degradation of the mutant proteins compared to the wild type proteins. Production levels of wild type MHAS2168, Rv1410, or their mutants expressed in *M. smegmatis* dKO using complementation vector pFLAG were probed by Western blotting via a C-terminal 3xFLAG tag. *M. smegmatis* dKO harboring the empty pFLAG vector (EV) served as a negative control to distinguish unspecific bands. (c) Size exclusion chromatography profiles of selected Rv1410 mutants purified from *E. coli* membranes show proper folding and insertion to the membrane. (d) SDS-PAGE analysis of Rv1410 mutants' monodisperse peaks from (c). Source data are provided in the Supplementary Source Data section.

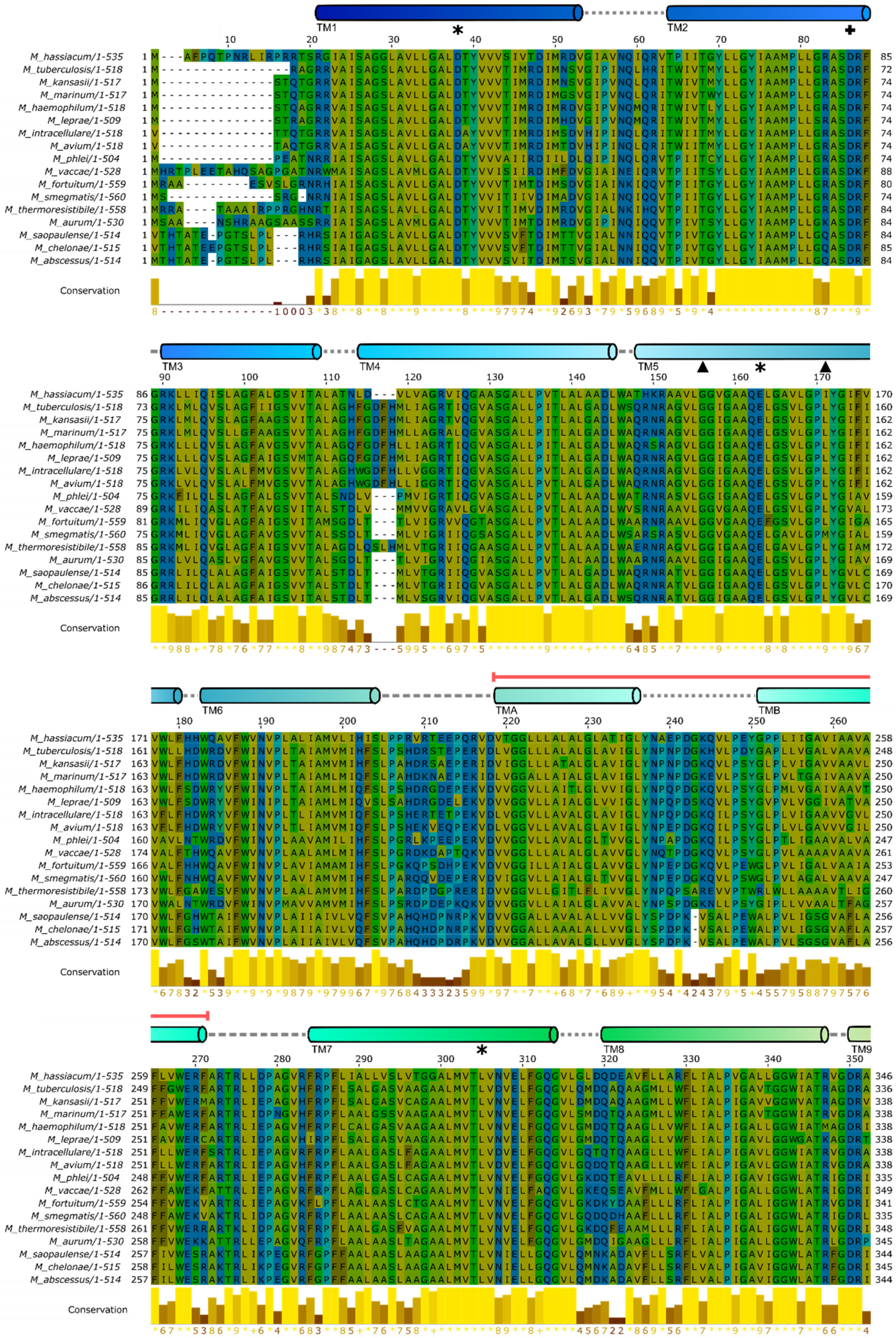

TM1
TM2
TM3
TM4
TM5
TM6
TMA
TMB
TM7
TM8
TM9
M_hassiacum/1-535
M_tuberculosis/1-518
M_kansasii/1-517
M_marinum/1-517
M_haemophilum/1-518
M_leprae/1-509
M_intracellulare/1-518
M_avium/1-518
M_phlei/1-504
M_vaccae/1-528
M_fortuitum/1-559
M_smegmatis/1-560
M_thermoresistibile/1-558
M_aurum/1-530
M_saopaulense/1-514
M_chelonae/1-515
M_abscessus/1-514
Conservation

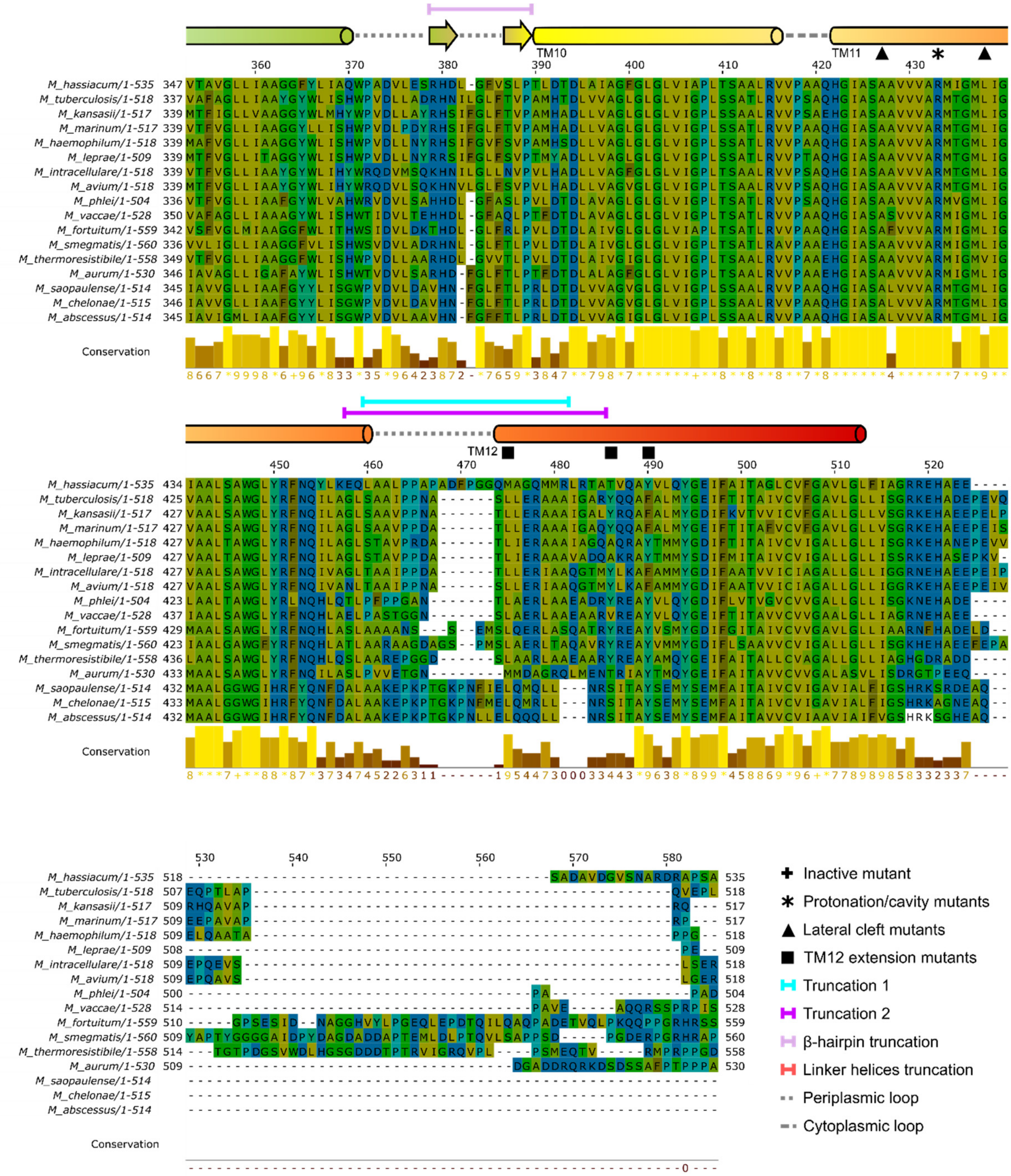

Supplementary Figure 5. Multiple sequence alignment (MSA) of primary structures of 17 mycobacterial Rv1410 homologues. The amino acid residues in the MSA are colored according to their hydrophobicity (hydrophilic residues – blue; hydrophobic residues – gold). Secondary structure elements corresponding to the primary structure are depicted above the MSA: transmembrane α-helices and β-sheets are colored in the rainbow color scheme with the N-terminal end being blue and C-terminal end being red. Loops between secondary structure elements are depicted as gray dashed lines (periplasmic loops – short dashes; cytoplasmic loops – long dashes). Point mutations analyzed in this study are marked by different symbols above the corresponding amino acid residue in the MSA as indicated. Different truncation mutants are marked with colored lines showing the extent of truncations as indicated.

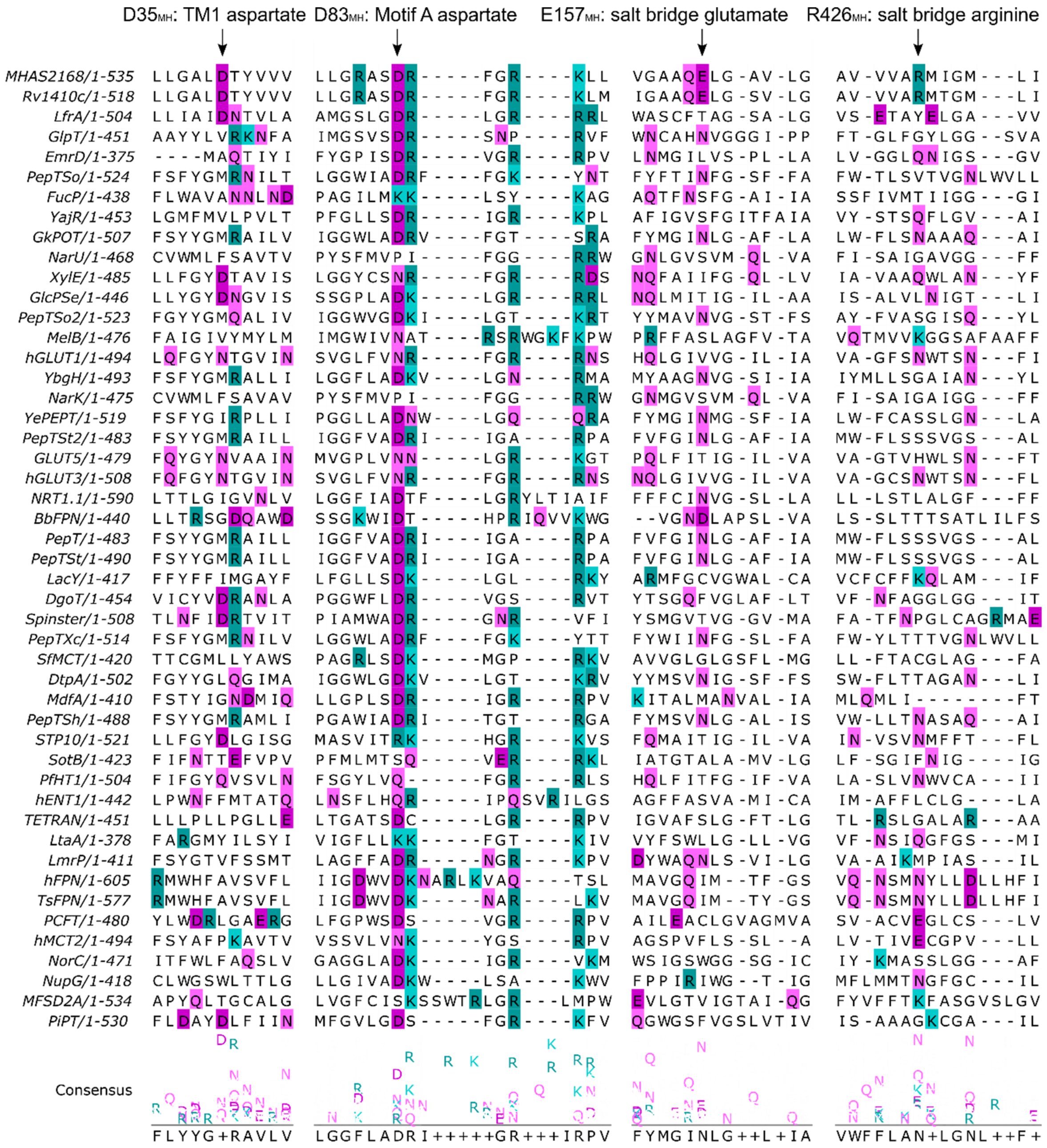

Supplementary Figure 6. Conservation of different residues potentially involved in proton coupling in Rv1410 and MHAS2168 among other MFS transporters, depicted by a multiple sequence alignment. Positively charged residues arginine and lysine are highlighted in cyan (R and K). Negatively charged residues glutamate and aspartate are highlighted in dark pink (E and D). Glutamine and asparagine are highlighted in light pink (Q and N). 1st block: D35$_{MH}$/D22$_{Mtb}$ (TM1, side chain within the N-domain) is mostly conserved in mycobacterial MFS transporters and some sugar porters. 2nd block: Motif A aspartate D83$_{MH}$/D70$_{Mtb}$ (cytoplasmic loop between TM2 and TM3) is very conserved among MFS transporters. 3rd block: Salt bridge glutamate E157$_{MH}$/E147$_{Mtb}$ (TM5, side chain within the central cavity) seems to be present only in mycobacterial TAG exporters MHAS2168 and Rv1410. 4th block: Salt bridge arginine R426$_{MH}$/R417$_{Mtb}$ (TM11, side chain within the central cavity) seems to be present only in mycobacterial TAG exporters MHAS2168 and Rv1410.

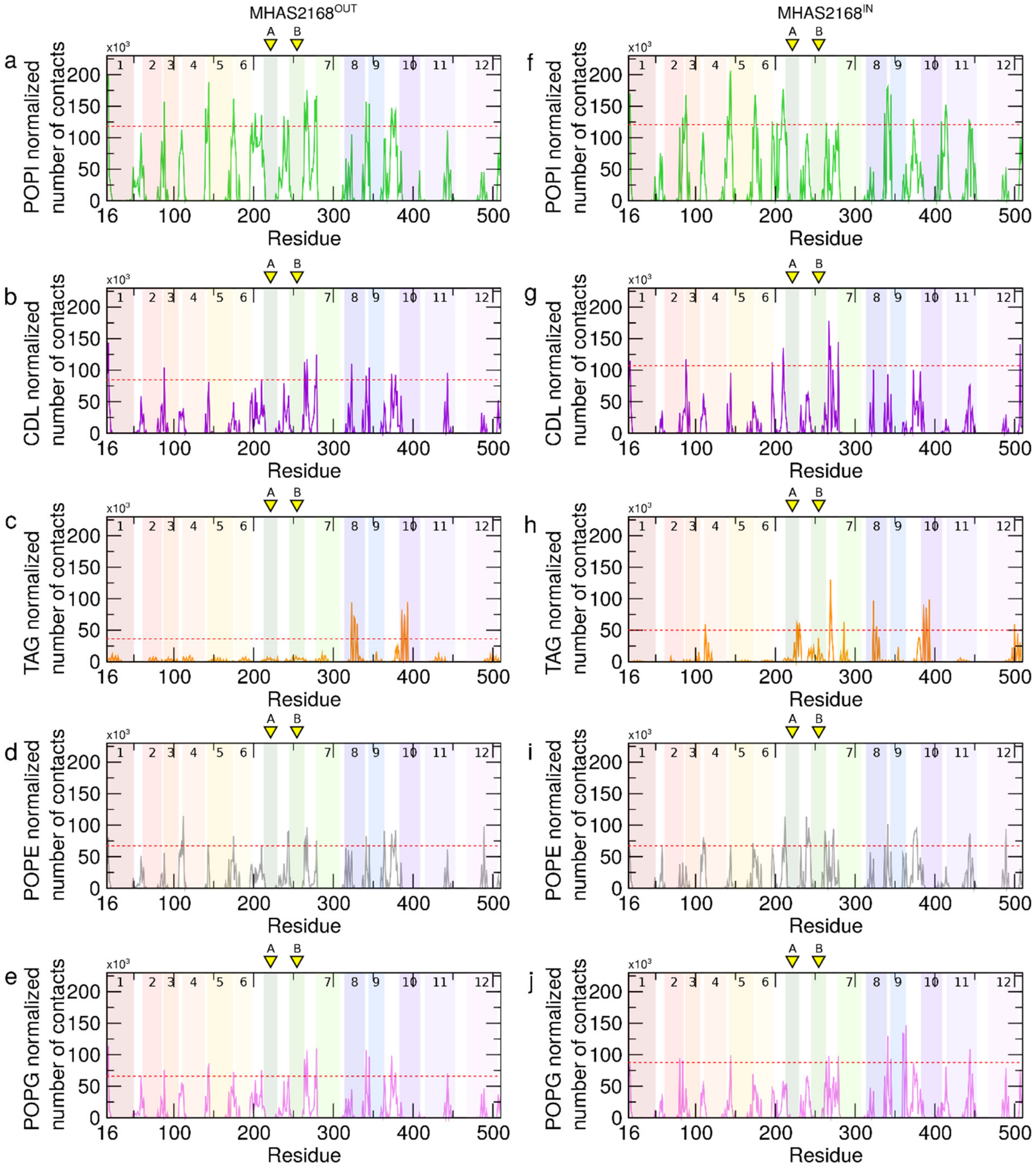

Supplementary Figure 7. Lipid interactions observed in the simulations. Protein-lipid contacts based on simulations performed with the MHAS2168^{OUT} structure (a)-(e) or the MHAS2168^{IN} homology model (f)-(j). The lipid color code is the same as in Fig. 3a. The transmembrane helices of MHAS2168 are numbered and depicted as rainbow-colored bars. Linker helices A and B are indicated with yellow arrows. The red dotted line is the threshold above which the contact has been considered as relevant.

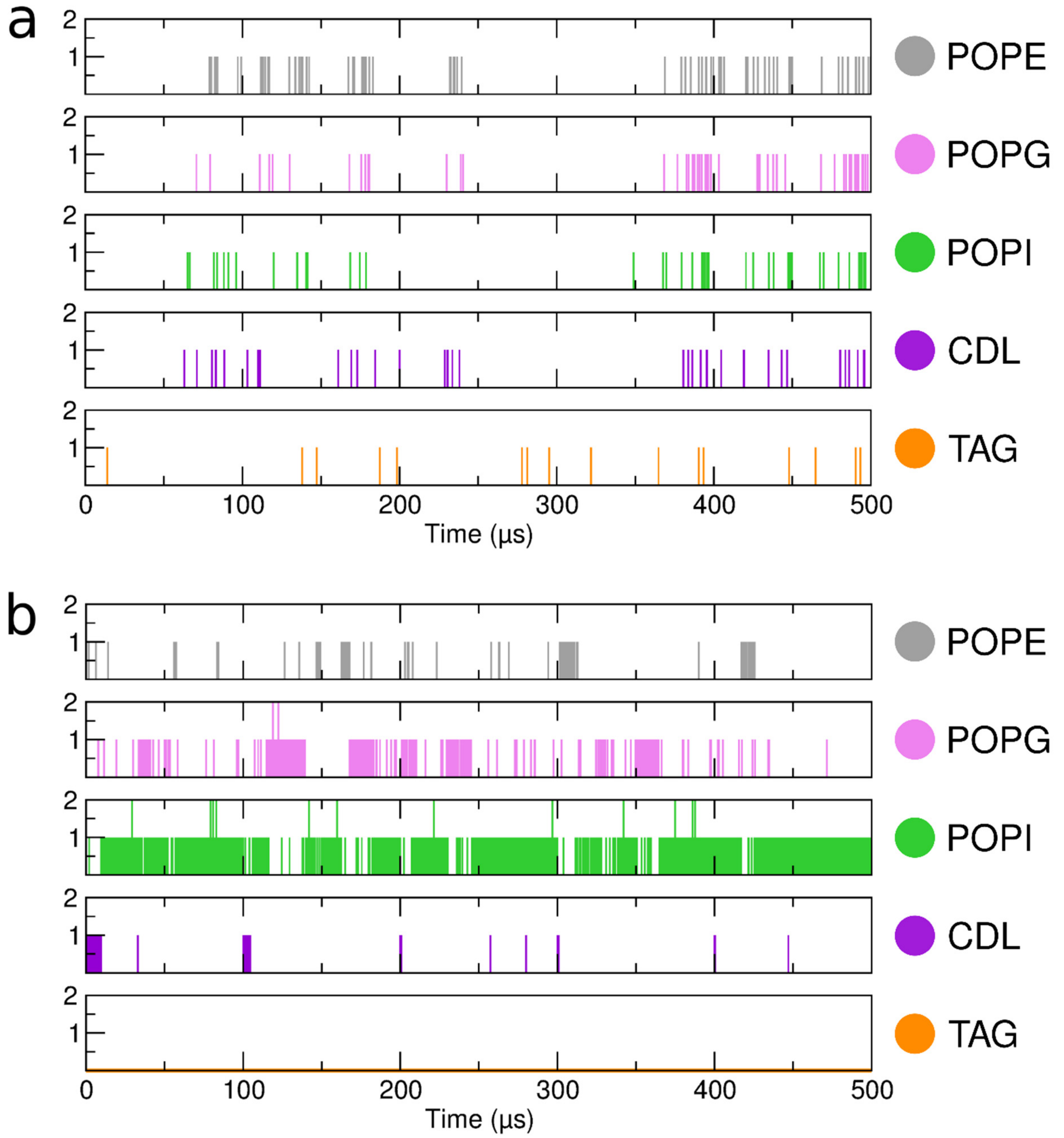

Supplementary Figure 8. Mapping TAG and phospholipid entrance from the membrane into the transporter central cavity during the MD simulations of MHAS2168. Number of entrance and snorkeling events of the membrane components within the protein central cavity during the MD simulation of the MHAS2168^{OUT} (a) and MHAS2168^{IN} (b) systems. An event of entrance/snorkeling was recorded if the phosphate bead or the TAG backbone bead overlapped with the cavity volume shown in Supplementary Fig. 9. All the five independent repeats of 100 µs each were concatenated together for clarity.

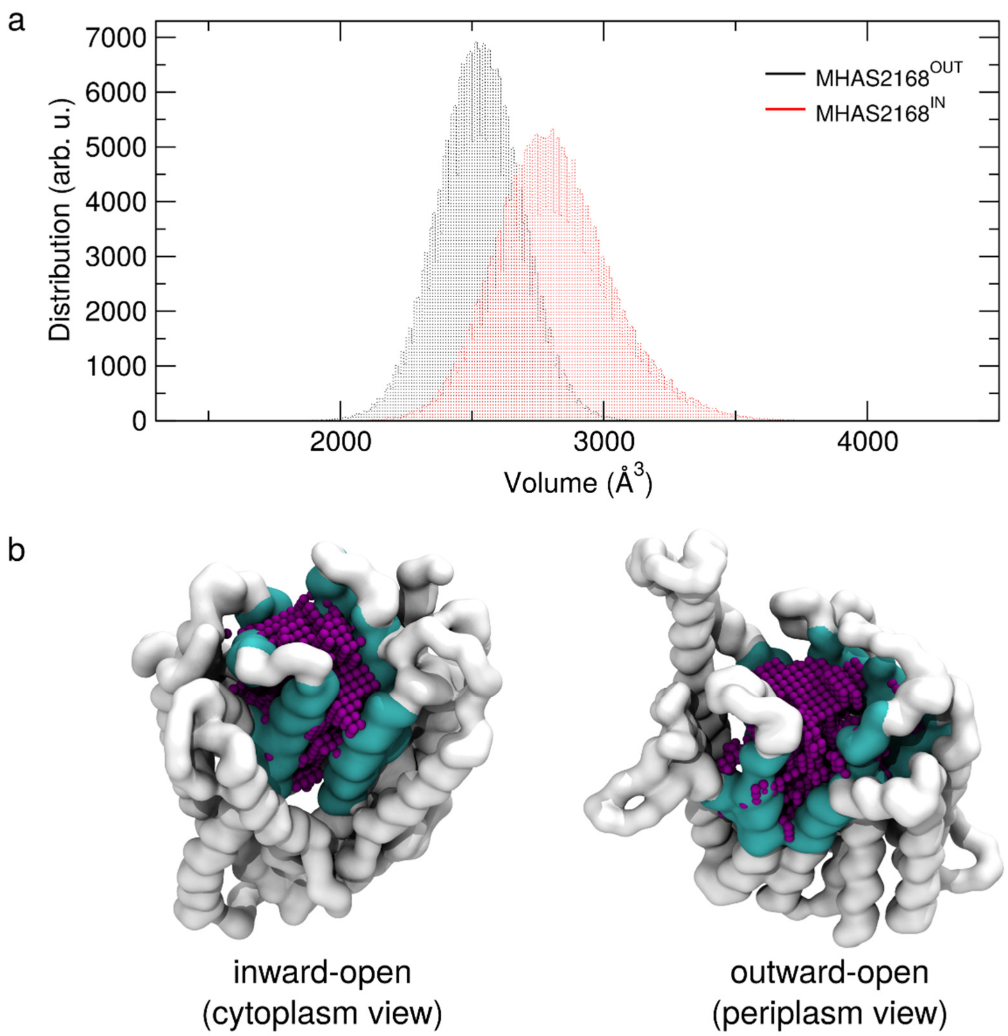

Supplementary Figure 9. The central cavity of MHAS2168. (a) The histograms show the volume of the central cavity encompassed by the N- and C-terminal domains calculated during the MD simulations of the outward-facing (the MHAS2168^{OUT} system) and the inward-facing (the MHAS2168^{IN} system) conformations. (b) Snapshots from the MD simulations with the central cavity and the protein backbone atoms highlighted in purple and white, respectively. Residues in cyan were selected for the cavity calculations (see Methods).

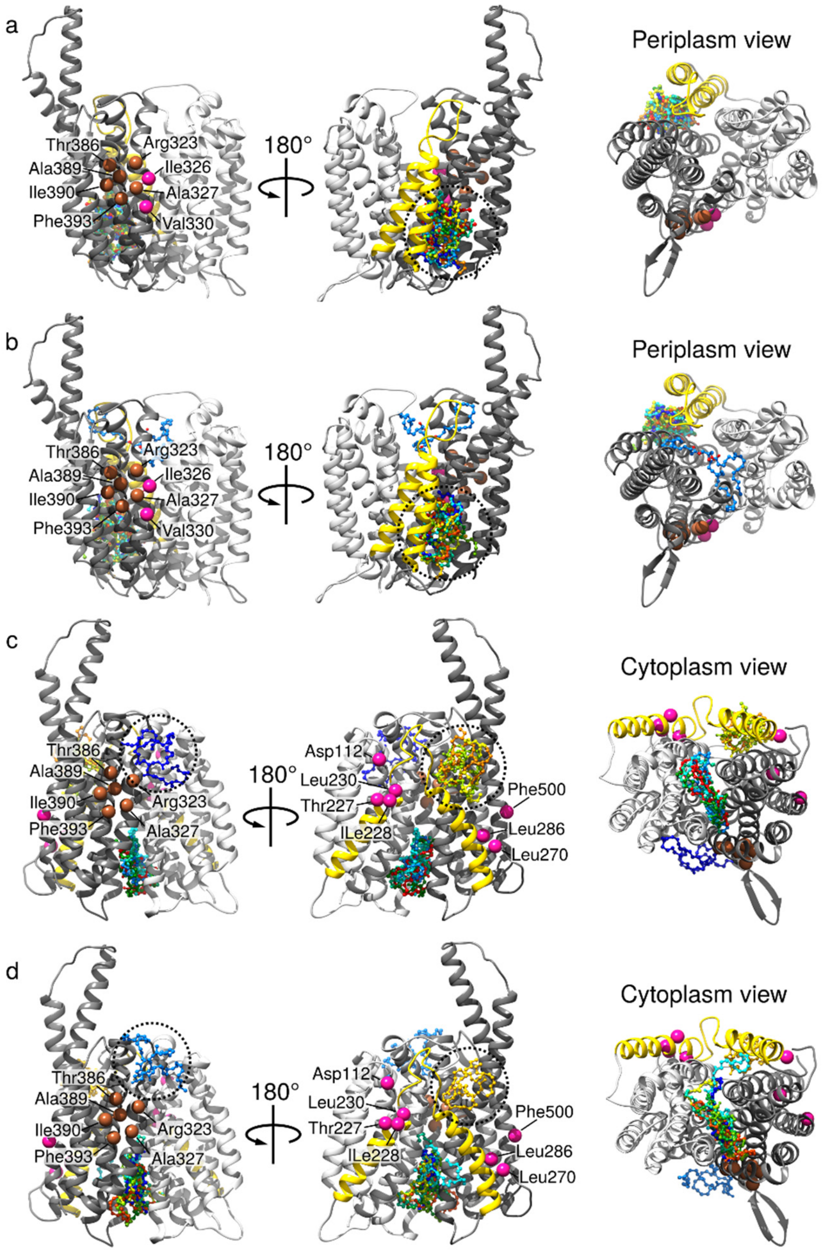

Supplementary Figure 10. Results of the molecular docking of TAG on the MHAS2168 structure. (a) Docking results for the MHAS2168 in outward-facing conformation (TAG is flexible; protein residues are rigid). The N- and C-terminal parts of the transporter are in ribbon and colored in light gray and dim gray, respectively. TMA and TMB linker helices are yellow. Docked TAG molecules are shown in ball and stick representations and colored rainbow from blue to red. The dotted circle indicates the docking hotspots. The Cα atoms of residues found in contact with TAGs in the CG-MD simulations are colored pink, while residues found in contact with TAGs in both the outward- and inward-facing conformations are colored brown (see Supplementary Table 4). (b) As in (a), but in addition to TAG, protein residues Glu157 and Arg426 were flexible during docking. (c) As in (a), but for MHAS2168 inward-facing conformation. (d) As in (a), but for MHAS2168 inward-facing conformation and, in addition to TAG, protein residues Glu157 and Arg426 were flexible during docking.

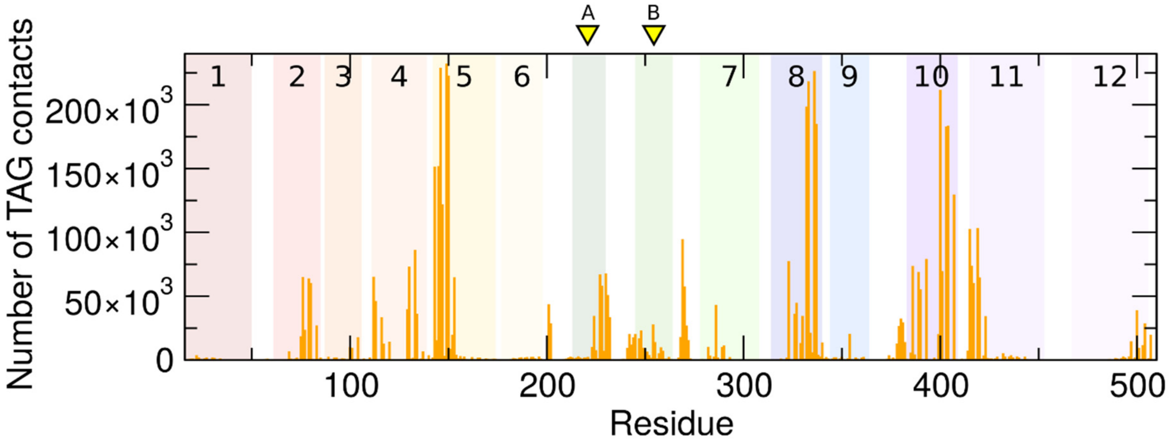

Supplementary Figure 11. MD simulations of inward-facing MHAS2168 with TAG initially placed within the central cavity. Overall protein-TAG contacts (orange) in the 135 μs of collected simulation time of MHAS2168$^{IN\text{-}TAG}$ (see text). The transmembrane helices of MHAS2168 are numbered and depicted as rainbow-colored bars. Linker helices TMA and TMB are indicated with yellow arrows.

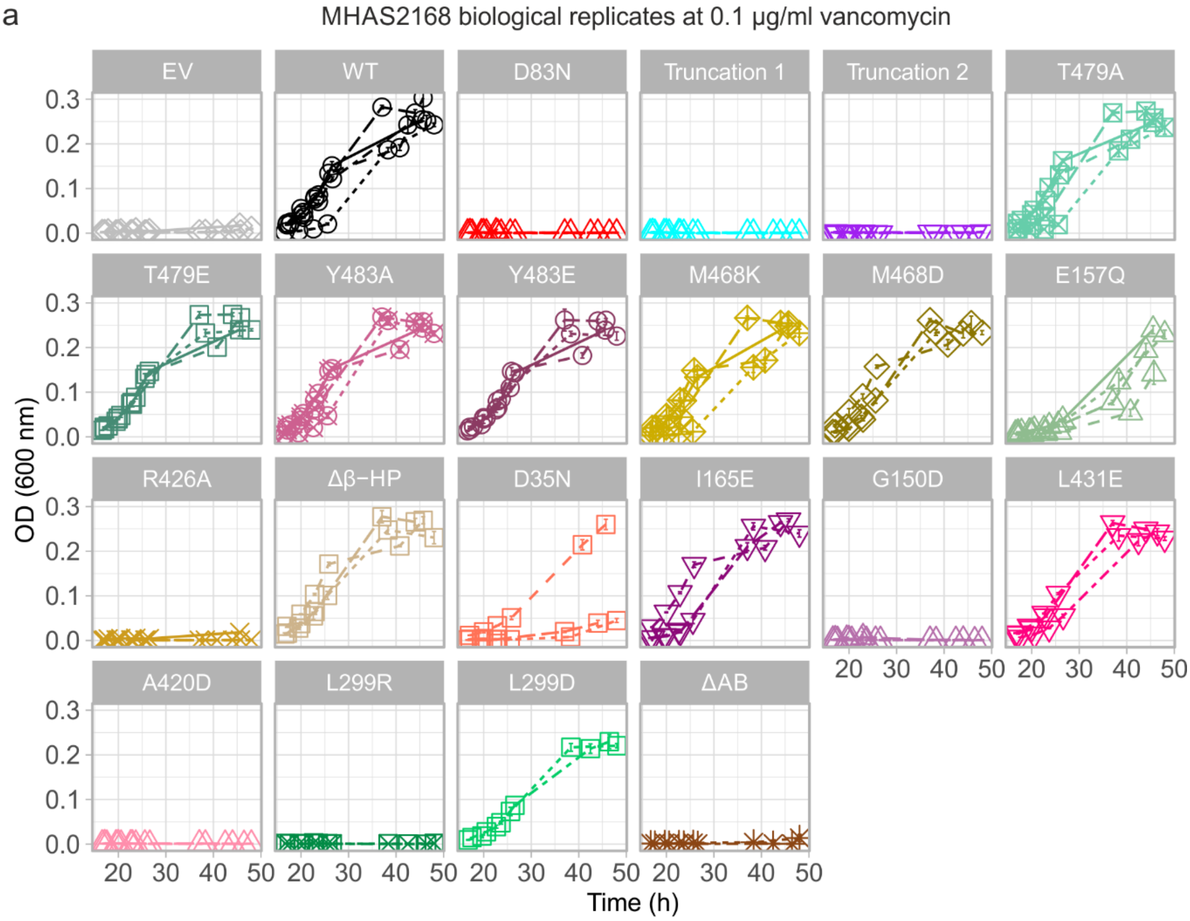
a
MHAS2168 biological replicates at 0.1 μg/ml vancomycin
EV
WT
D83N
Truncation 1
Truncation 2
T479A
T479E
Y483A
Y483E
M468K
M468D
E157Q
R426A
Δβ−HP
D35N
I165E
G150D
L431E
A420D
L299R
L299D
ΔAB
OD (600 nm)
Time (h)

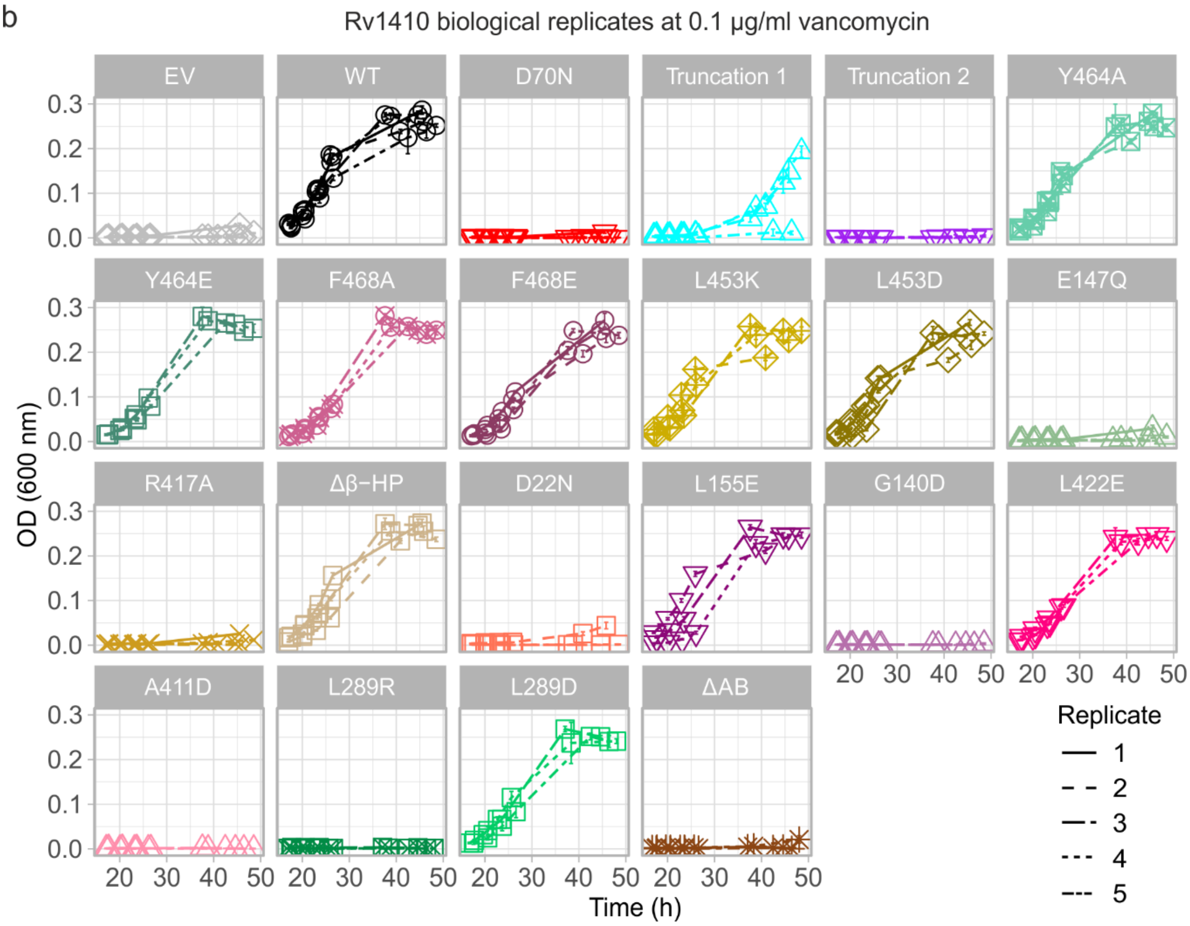
b
Rv1410 biological replicates at 0.1 μg/ml vancomycin
EV
WT
D70N
Truncation 1
Truncation 2
Y464A
Y464E
F468A
F468E
L453K
L453D
E147Q
R417A
Δβ−HP
D22N
L155E
G140D
L422E
A411D
L289R
L289D
ΔAB
OD (600 nm)
Time (h)
Replicate
1
2
3
4
5

c

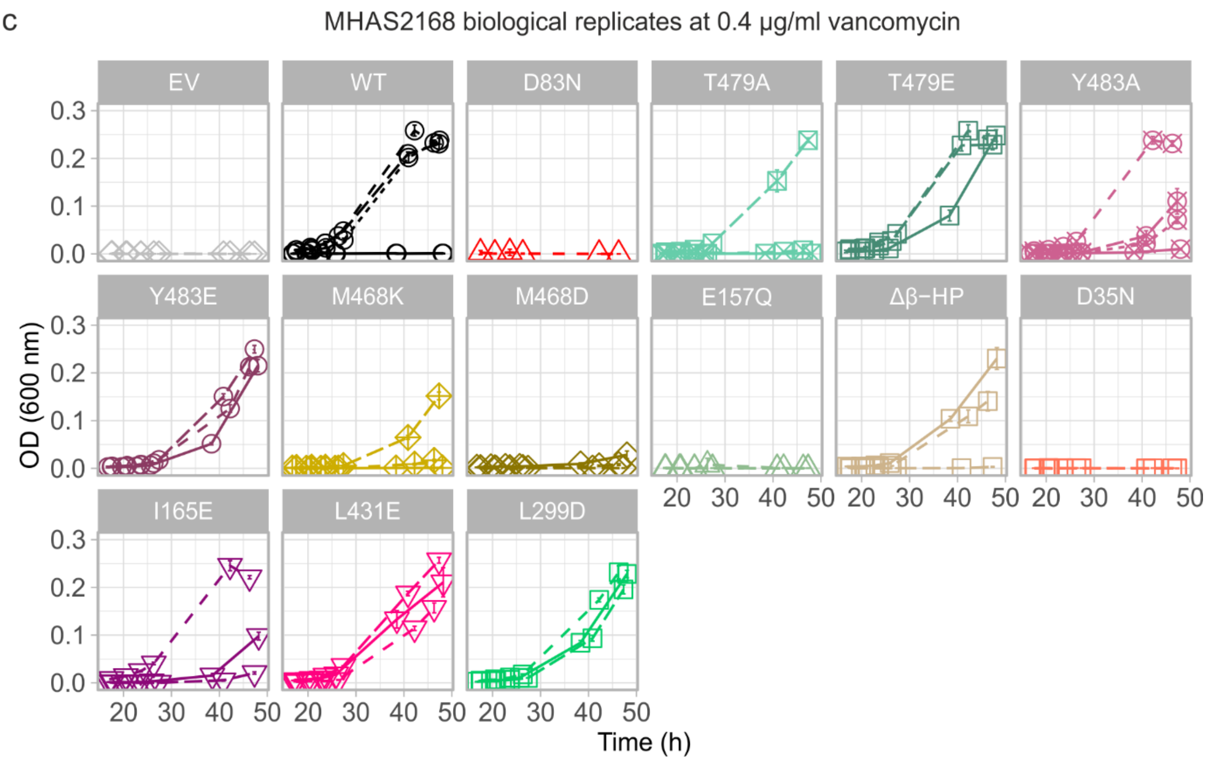

d

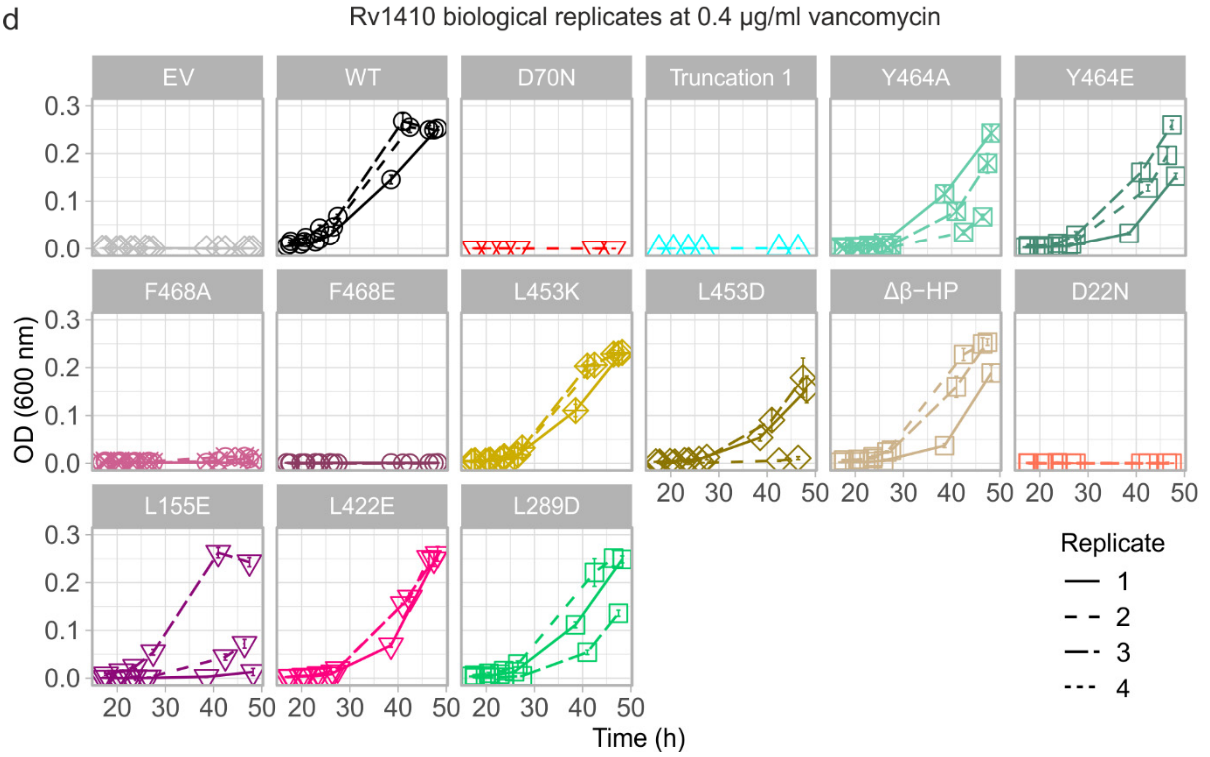

Supplementary Figure 12. All vancomycin sensitivity assay results at 0.1 and 0.4 μg/ml vancomycin concentration. Vancomycin sensitivity assays in *M. smegmatis* dKO cells, complemented with empty vector control (EV), wild type LprG/Rv1410 or MHAS2167/68 operon (WT), or mutant operons where LprG (Rv1411/MHAS2167) is intact, but the transporter Rv1410/MHAS2168 exhibits mutations as indicated. For each tested mutant, growth curves from all biological replicates (3-5) grown at 0.1 μg/ml (a)-(b) or 0.4 μg/ml (c)-(d) vancomycin concentration are shown as means. The error bars of the growth curves denote the standard deviation of four technical replicates. (a) and (c) MHAS2168 mutants and controls. (b) and (d) Rv1410 mutants and controls.

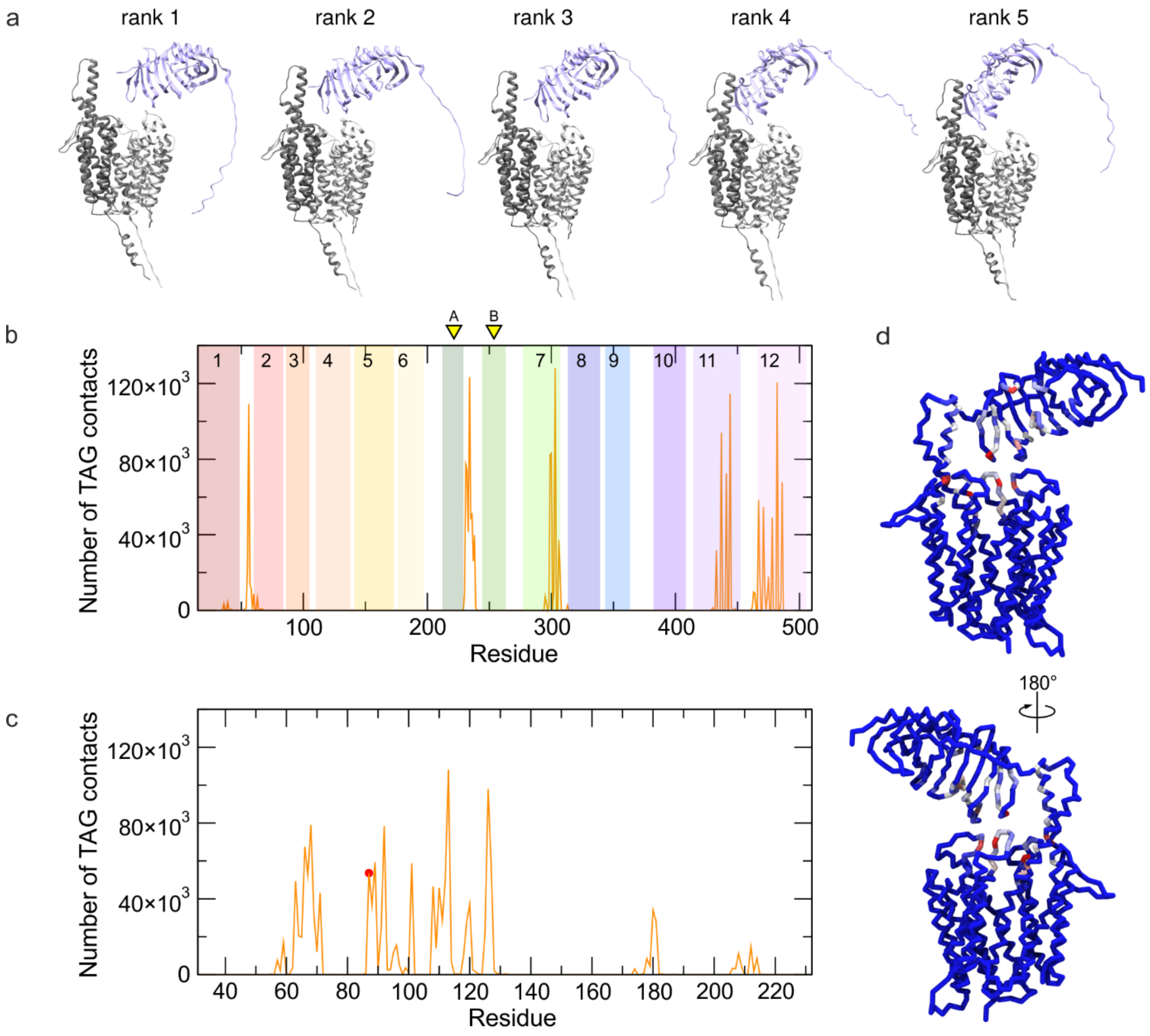

Supplementary Figure 13. Analysis of TAG contacts with MHAS2168^{OUT}-LprG during MD simulations. (a) Five possible models of the MHAS2168^{OUT}-LprG complex predicted by ColabFold. The rank 2 model was selected for the MD simulations. (b) Average number of MHAS2168-TAG contacts among the five repeat simulations (orange). The transmembrane helices of MHAS2168 are numbered and depicted as rainbow-colored bars. Linker helices TMA and TMB are indicated with yellow arrows. (c) LprG-TAG contacts (orange). T87, which corresponds to V91 in *M. tuberculosis* LprG, is indicated in red. (d) TAG contacts projected onto the protein backbone and colored from blue (no contacts) to red (large number of contacts).

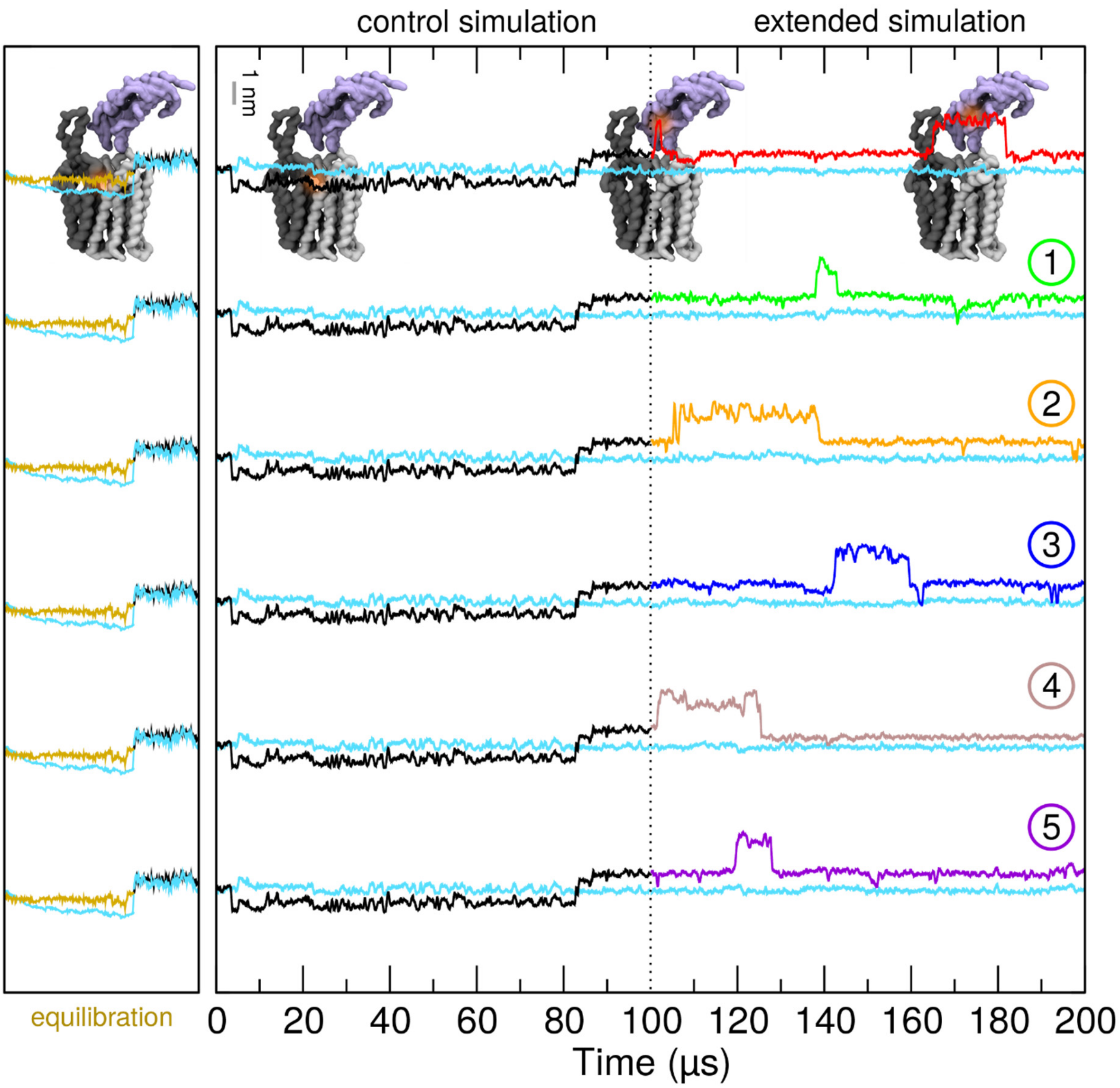

Supplementary Figure 14. TAG transfer into the LprG hydrophobic cavity in the control MD simulations of the MHAS2168^{OUT}-LprG complex. A control simulation was carried out for 100 µs, starting with TAG in a 2-tails-down configuration. The black line depicts the z-coordinate of the center of mass of the TAG molecule. The cyan line is the z-coordinate of the average center of mass of the phosphate groups of the periplasmic membrane leaflet. The extended simulation (in red), and the five repeat simulations with different starting velocities (green, orange, blue, grey and purple) are indicated and numbered respectively. The gold line is the equilibration phase (see Methods). The insets of the MHAS2168^{OUT}-LprG complex shown in the top panel indicate the position of the TAG (orange shade).

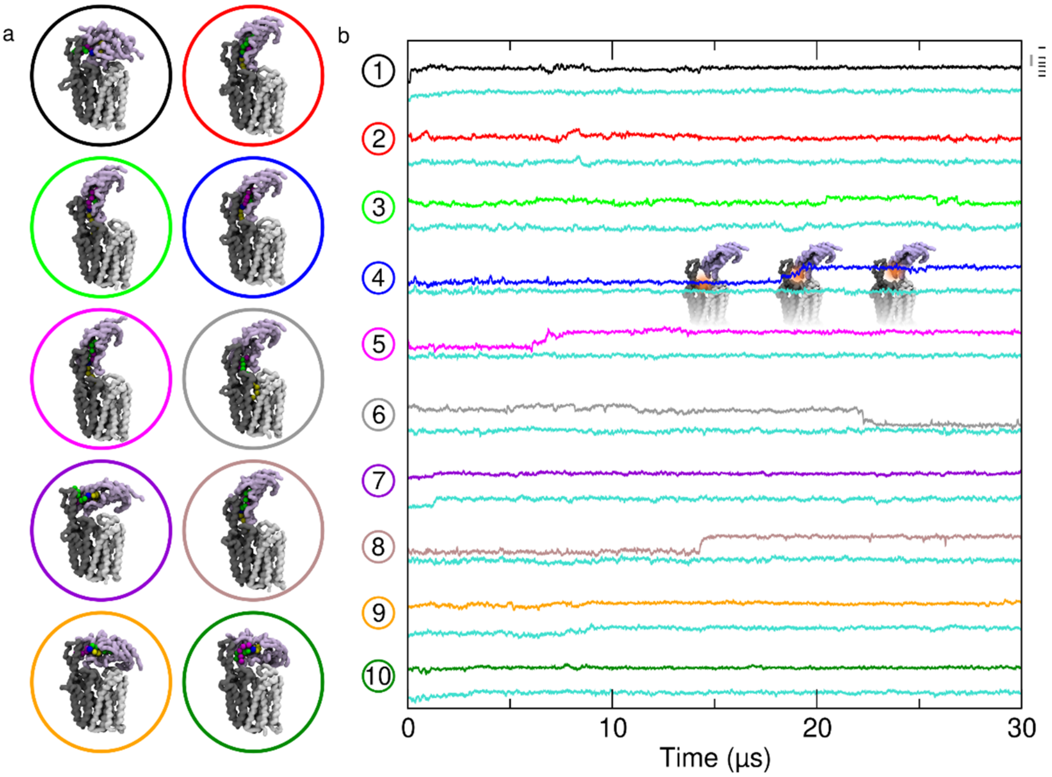

Supplementary Figure 15. TAG transfer into LprG in the control MD simulations of the MHAS2168^OUT-LprG complex without MHAS2168-LprG elastic network bonds. (a) Final snapshots of the ten control simulations (consecutively numbered) carried out for 30 μs each and with different starting velocities. Simulations started with TAG in a 2-tails-up configuration and after the equilibration phase of the MHAS2168^OUT-LprG system. (b) The cyan line is the z-coordinate of the average center of mass of the phosphate groups of the upper membrane leaflet. The colored lines (as in (a)), depict the z-coordinate of the center of mass of the TAG molecule. The inset models of the MHAS2168^OUT-LprG complex shown in the panel indicate the position of the TAG (orange shade).

Supplementary Tables

Supplementary Table 1. X-ray data collection and refinement statistics. In parentheses, parameters of the highest resolution shell are shown.

	MHAS2168 + Nb_H2	
**Data collection**	**Crystal I (Full dataset)**	**Crystals II and III (Merged dataset)**
Space group	P 1 2_1 1	P 1 2_1 1
Cell dimensions		
a, *b*, *c* (Å)	57.75, 160.68, 82.78	57.78, 160.7, 82.96
α, β, γ (°)	90.0, 108.854, 90.0	90.0, 109.014, 90.0
Resolution (Å)	2.7 (2.76-2.70)	2.7 (2.76-2.70)
R_{meas} (%)	6.2 (124.1)	10.7 (183.8)
I/σ (I)	11.44 (0.97)	8.77 (0.97)
Completeness (%)	94.9 (97.8)	99.3 (99.7)
cc(1/2)	99.9 (46.7)	99.9 (34.7)
**Refinement**		
Resolution range (Å)		44.7 - 2.7 (2.797 - 2.7)
No. unique reflections		39031 (3911)
R-work/R-free		0.2450/0.2915
No. of atoms		
Macromolecules		8824
Ligands		0
Solvent		0
Average B-factor		92.85
RMS deviations		
Bonds		0.003
Angles		0.73
Ramachandran favored (%)		98.29
Ramachandran allowed (%)		1.71
Ramachandran outliers (%)		0
Rotamer outliers (%)		0.23
Clashscore		2.00
RSRZ (%)		14.4
MolProbity score		0.77

Supplementary Table 2. Cryo-EM data collection and processing statistics.

	**MHAS2168 + Mb_H2**	**Rv1410 + Mb_F7**
**Data collection and processing**		
Magnification	130 000x	130 000x
Voltage (kV)	300	300
Electron exposure ($e^-/Å^2$)	66.54	65.0 / 55.0
Defocus range (µm)	-1 to -2.5 µm	-1 to -2.5 µm
Pixel size (Å)	0.325 (in super-resolution)<br>0.65 (in construction)	0.325 (in super-resolution)<br>0.65 (in construction)
Symmetry imposed	C1	C1
No. of initial particle images	4 759 395	1 833 683
No. of final particle images	402 229	127 196
Map resolution (Å)	3.99	7.51
FSC threshold	0.143	0.143

Supplementary Table 3. MD simulations. 1-palmitoyl-2-oleyl-phosphatidylethanolamine, POPE; 1-palmitoyl-2-oleyl-phosphatidylglycerol, POPG; cardiolipin, CDL; 1-palmitoyl-2-oleyl-phosphatidylinositol, POPI; triacylglycerol, TAG. ul = upper leaflet; ll = lower leaflet. ‡ = The systems with 2 and 1 TAG hydrophobic tails pointing upwards (towards LprG) as well as the control simulations where all the intermolecular harmonic potential elastic bonds within the complex were removed, are identical. The Martini model for the cardiolipin has minus 2 charge (both the phosphatidyl groups are charged, namely CDL2). The Martini model for TAG represents a glycerol with three C18:1 oleoyl tails (namely TOG[83]).

System	Protein particles	Lipids	Membrane composition (leaflet %)	Water particles	Ions (Na^+, Cl^-)	Total particles	Box size (nm^3)
MHAS2168^{OUT}	985	ul: 100 molecules<br>ll: 100 molecules<br>2942 particles	POPE: 34<br>POPG: 29<br>CDL: 15<br>POPI: 21<br>TAG: 1	5631	222, 63	9843	ca. 1137
MHAS2168^{IN}	985	ul: 100 molecules<br>ll: 98 molecules<br>2918 particles	POPE: 34<br>POPG: 29<br>CDL: 15<br>POPI: 21<br>TAG: 1	5850	224, 66	10043	ca. 1166
MHAS2168^{IN-TAG}	985	ul: 100 molecules<br>ll: 98 molecules<br>2918 particles	POPE: 34<br>POPG: 29<br>CDL: 15<br>POPI: 21<br>TAG: 1	5850	224, 66	10043	ca. 1166
MHAS2168^{OUT}-LprG‡	1384	ul: 196 molecules<br>ll: 189 molecules<br>5649 particles	POPE: 34<br>POPG: 29<br>CDL: 15<br>POPI: 21<br>TAG: 1	8798	493, 169	16493	ca. 1872
Myco^{mem}	-	ul: 141 molecules<br>ll: 141 molecules<br>4142 particles	POPE: 34<br>POPG: 29<br>CDL: 15<br>POPI: 21<br>TAG: 1	5702	329, 103	10276	ca. 1175
MHAS2168$^{OUT-TAG7}$	985	ul: 100 molecules<br>ll : 100 molecules<br>2956 particles	POPE: 32<br>POPG: 27<br>CDL: 14<br>POPI: 20<br>TAG: 7	5599	212, 63	9815	ca. 1140
MHAS2168$^{IN-TAG7}$	985	ul: 100 molecules<br>ll : 100 molecules<br>2956 particles	POPE: 32<br>POPG: 27<br>CDL: 14<br>POPI: 20<br>TAG: 7	5566	212, 63	9782	ca. 1120

Supplementary Table 4. Protein-lipid interactions in CG-MD simulations.

**MHAS2168 interactions with TAG [a] (threshold of 40%)**						
**Phe393**	**Thr386**	**Ala327**	**Arg323**	**Ala389**	**Ile390**	Ile326
Val330	Leu270	Leu230	Phe500	Leu286	Thr227	Asp112
Ile228						
**MHAS2168 interactions with POPE [b] (threshold of 60%)**						
**Asp112**	**Glu489**	**Arg267**	**Phe378**	**Arg341**	**Tyr244**	**Glu243**
**Arg373**	**Asn110**	**Gly377**	**His374**	Phe265	Arg18	His175
Arg279	Arg264	Gln364	His143	Arg345	Asp212	Gln239
Pro242	Pro273	Trp262	Asp375	Leu376	Arg444	Glu263
Asp272	Tyr443	Leu241	Ser17	Trp172	Val213	Gln58
Asn232						
**MHAS2168 interactions with POPG [c] (threshold of 60%)**						
**Arg279**	**Arg341**	**Arg267**	**Arg345**	**Arg264**	**Lys144**	Arg18
Arg373	His143	Thr16	Lys88	Arg210	His175	Phe378
Tyr443	Phe265	Gln364	Tyr360	Leu361	Arg444	Arg80
Arg84						
**MHAS2168 interactions with POPI [d] (threshold of 60%)**						
**Arg18**	**Lys144**	**Ser17**	**Thr16**	**Arg279**	**His175**	**Arg341**
**Lys88**	**Arg264**	**Arg345**	**Arg373**	**Trp140**	**His143**	**Arg202**
**Arg210**	Arg267	Arg277	Phe378	Gly377	Lys238	His374
Glu243	Phe278	Phe265	Ser198	Gln209	Thr340	Gln414
Phe174	Ala413	Trp337	Gly510	Arg87	Val211	Arg84
His415	His176	Tyr443	His196	Arg408	Pro208	Arg444
Val342						
**MHAS2168 interactions with CDL [e] (threshold of 60%)**						
**Arg18**	**Arg279**	**Thr16**	**Arg267**	Arg264	Arg323	Lys88
Arg345	Tyr443	Ser17	Arg373	Phe378	Arg341	Phe507
Arg269	Arg210	Thr268	His196			
**MHAS2168 interactions with TAG in the MHAS2168-LprG complex [f]**						
**Gln56**[1-5]	**Glu234**[1-5]	**Glu303**[1-5]	**Leu437**[1-5]	**Arg444**[1-5]	**Ala482**[1-5]	Tyr231[1,2,5]
Asn232[2,3,5]	Ala233[3]	Pro235[1,2,5]	Asp236[1,5]	Leu299[1,2,5]	Val300[1,3,5]	Gly306[2]
Gly441[1,3,5]	Gln467[1,4]	Gln471[1,4]	Ala478[3]	Gln486[1,3,5]		
**LprG interactions with TAG in the MHAS2168-LprG complex [g]**						
Leu63[1,3]	Leu66[1,3,4]	Pro67[1,4]	Ile68[1,3,4,5]	Leu71[4]	Thr87[1,4]	Thr88[4]

Val89^{1,4}	Met92^{2,3,5}	Phe101^{1,4}	Leu108^{1,4}	Ala110^{4}	Leu112^{4}	Phe113^{1,2,3,5}
Ile120^{4}	Ile126^{1,3,4,5}	Tyr127^{1,3,4}				
**MHAS2168 interactions with TAG in the MHAS2168^{IN-TAG} system [h]**						
Leu149	Ala146	Gly150	Leu333	Gly336	Ala400	Arg323
Trp337	Thr403	Ser404	His143	Arg145	Ala147	Leu407
Arg269	His415	Ser419				

(*a-e*) The residues listed have lipid contacts above the indicated threshold with respect to the residue that has the maximum number of contacts for that specific lipid. Residues underlined and not underlined refer to MHAS2168^{IN} and MHAS2168^{OUT}, respectively. Residues highlighted in bold are found in both MHAS2168^{IN} and MHAS2168^{OUT}. (*f*) The residues listed have TAG contacts above a threshold of 40% with respect to the residue that has the maximum number of TAG contacts. The superscript indicates in which repeat simulation the interaction was found. This is because TAG samples the MHAS-LprG regions within the hydrophobic tunnel for different time periods in different repeat simulations (see Fig. 6), and thus statistics for that region is improved in the respective repeat(s). Residues highlighted in bold are found in all the repeat simulations. (*g*) The residues listed have TAG contacts above a threshold of 40% with respect to the residue that has the maximum number of TAG contacts. The superscript indicates in which repeat simulation the interaction was found (see also *f*). (*h*) The residues listed have TAG contacts above a threshold of 40% with respect to the residue that has the maximum number of TAG contacts.

Supplementary Table 5. Primers used for generating pFLAG plasmids with shuffled operons.

**Primer name**	**Sequences (5′ -> 3′)**
**FX cloning *M. hassiacum* ORFs**	SapI recognition site and scar are underlined
MHASS1410_for	ATATATGCTCTTCTAGTGCTTTCCCGCAGACACCGAACCGACTG
MHASS1410_rev	ACTGACGCTCTTCATGCAGCTGATGGCGCGCGGTCTCGAGC
FX_MHAS2167_FOR	ATATATGCTCTTCTAGTCAGACCCGCCTGACGGCGATCCTCGCC
FX_MHAS2168_REV	TATATAGCTCTTCATGCCGCTGATGGCGCGCGGTCTCGAGCGTT
**CPEC pFLAG_*MHAS2167***	
pFLAG_MHAS2167_FOR2	CGAGGCATGCGAAGGAGATATACATATGCAGACCCGCCTGACGG
FLAG_MHAS2167_REV2	ACCGTCATGGTCTTTGTAGTCTGCGGCGGCCGGCTTG
FLAG_FOR	GCAGACTACAAAGACCATGACGGT
pFLAG_REV2	CATATGTATATCTCCTTCGCATGCCTCG
**CPEC pFLAG_*MHAS2167_Rv1410***	
pFLAG_MHAS2167_FOR	GGCATGCGAAGGAGATATACATATGAGTAGTCAGACCCGCCTGACG
FLAG_RV1410_REV	ACCGTCATGGTCTTTGTAGTCTGCTGCGAGCGGCTCCACTTG
FLAG_FOR	GCAGACTACAAAGACCATGACGGT
pFLAG_REV	ACTCATATGTATATCTCCTTCGCATGCC
**CPEC pINIT_*Rv1411_mfs***	
pINIT_FOR2	GAAGCCCTGGGCCAACTTTTG
Rv1411_REV	GCTGATCAGCTCACCGGGG
pINIT_REV2	CAAAAGTTGGCCCAGGGCTTC
Rv1411_MSM3069_FOR	CCCCGGTGAGCTGATCAGCGTGAGTTCCCGGGGCAACC
Rv1411_MHAS2168_FOR	CCCCGGTGAGCTGATCAGCATGGCGTTCCCGCAGACAC
Rv1411_MAB2807_FOR	CCCCGGTGAGCTGATCAGCGTGACGCACACAGCGACG
**CPEC pINIT_*lprG_Rv1410***	
pINIT_REV2	CAAAAGTTGGCCCAGGGCTTC
Rv1410_FOR	TCAGCATGCGAGCAGGACG
pINIT_FOR2	GAAGCCCTGGGCCAACTTTTG
Rv1410_MAB2806_REV	CGTCCTGCTCGCATGCTGATCACTGGGCGGGCTTGTC
Rv1410_MSMEG3070_REV	CGTCCTGCTCGCATGCTGATCAGGCCGCGGGCTTG
Rv1410_MHAS2167_REV	CGTCCTGCTCGCATGCTGATCAGGCGGCCGGCTTG

Supplementary Table 6. Primers used for introducing mutations into Rv1410 and MHAS2168.

**Primer name**	**Sequences (5′ -> 3′)**
**Rv1410**	
A411D_FOR	GCATCGCTTCGGACGCGGTGGTGGTC
A411D_REV	GCGTCCGAAGCGATGCCGTG
D22N_FOR	GGC GCC CTG AAC ACC TAT GTC GTG
D22N_REV	AGG TGT TCA GGG CGC CCA G
E147Q_FOR	GCCGCGCAGCAGCTCGGCAG
E147Q_REV	CTGCCGAGCTGCTGCGCGG
F468A_FOR	GTACCAGCAGGCGGCCGCGCTGATGTAC
F468A_REV	GTACATCAGCGCGGCCGCCTGCTGGTAC
F468E_FOR	GTACCAGCAGGCGGAGGCGCTGATGTAC
F468E_REV	GTACATCAGCGCCTCCGCCTGCTGGTAC
G140D_FOR	GACGGTATCGGCGCCGCGCAGGAG
G140D_REV	GCGCCGATACCGTCGAGCAC
L155E_FOR	CTG GGC CCG GAG TAC GGA ATC TTC ATC GTT TG
L155E_REV	CCG TAC TCC GGG CCC AGA ACG CTG
L289D_FOR	CGCTGATGGTGACGGACGTTGATGTCGAGCTGTTC
L289D_REV	CGAACAGCTCGACATCAACGTCCGTCACCATCAG
L289R_FOR	CTGATGGTGACGCGCGTTGATGTCGAGCTG
L289R_REV	CAGCTCGACATCAACGCGCGTCACCATCAG
L422E_FOR	GGATGACCGGCATGGAGATCGGCGTG
L422E_REV	CCACGCCGATCTCCATGCCGGTCATC
L453D_FOR	CCCAACGCCAGCGACCTCGAGCGCGC
L453D_REV	GCGCGCTCGAGGTCGCTGGCGTTGGG
L453K_FOR	CCCAACGCCAGCAAGCTCGAGCGCGC
L453K_REV2	GAGCTTGCTGGCGTTGGGCGGGAT
R417A_FOR	GGTGGTGGTCGCCGCGATGACCGGCATG
R417A_REV	CATGCCGGTCATCGCGGCGACCACCAC
Y464A_FOR	CAATTGGAGCCCGGGCCCAGCAGGCGTTC
Y464A_REV	GAACGCCTGCTGGGCCCGGGCTCCAATTG
Y464E_FOR	CAATTGGAGCCCGGGAGCAGCAGGCGTTC
Y464E_REV	GAACGCCTGCTGCTCCCGGGCTCCAATTG

Rv_delAB_FOR	GCTCGCACCCGGCTG
Rv_delAB_REV	CAGCCGGGTGCGAGCGTCGACTCGCTCGGGCTC
Rv_delbHP_REV	CATCGCACCACCACCACCATCGGCCAACAGGTCCAC
Rv_delbHP_FOR	GGTGGTGGTGGTGCGATGCACACCGACCTG
**MHAS2168**	
A420D_FOR	CATCGCCTCGGACGCCGTGGTGGTG
A420D_REV	CACCACCACGGCGTCCGAGGCGATG
D35N_FOR	GCG CGC TGA ACA CCT ACG TCG TG
D35N_REV	AGG TGT TCA GCG CGC CGA G
D83N_FOR	GCCGGGCGTCCAACCGGTTC
D83N_REV	GTTGGACGCCCGGCCCAG
E157Q_FOR	CGGCCCAGCAGCTCGGTGCCGTG
E157Q_REV	CGAGCTGCTGGGCCGCACCGAC
G150D_FOR	CGGTGCTCGACGGAGTCGGTG
G150D_REV	CACCGACTCCGTCGAGCAC
I165E_FOR	GTG CTC GGC CCC GAG TAC GGC ATC TTC GTG
I165E_REV	GTA CTC GGG GCC GAG CAC GGC AC
L299D_FOR	CGCTGATGGTGACCGACGTCAACGTCGAACTGTTC
L299D_REV	CCGAACAGTTCGACGTTGACGTCGGTCACCATCAG
L299R_FOR	CTGATGGTGACCCGCGTCAACGTCGAACTGTTC
L299R_REV	GAACAGTTCGACGTTGACGCGGGTCACCATCAG
L431E_FOR	GGCATGGAAATCGGCATCGCCGCGCTGAG
L431E_REV	CGGCGATGCCGATTTCCATGCCGATCATG
M468D_FOR	CGGGTGGCCAGGACGCCGGCCAGATGATG
M468D_REV	CTGGCCGGCGTCCTGGCCACCCGGAAAG
M468K_FOR	GTGGCCAGAAGGCCGGCCAGATG
M468K_REV	CATCTGGCCGGCCTTCTGGCCAC
R426A_FOR	GTGGTGGTGGCGGCCATGATCGGCATGCTG
R426A_REV	GCATGCCGATCATGGCCGCCACCACCAC
T479A_FOR	CTGCGCACCGCCGCGGTCCAGGCCTAC
T479A_REV	GTAGGCCTGGACCGCGGCGGTGCGCAG
T479E_FOR	CTGCGCACCGCCGAGGTCCAGGCCTAC
T479E_REV	GTAGGCCTGGACCTCGGCGGTGCGCAG
Y483A_FOR	

Y483A_REV	CACCGTCCAGGCCGCCGTGCTGCAGTAC
Y483E_FOR	GTACTGCAGCACGGCGGCCTGGACGGTG
Y483E_REV	CACCGTCCAGGCCGAGGTGCTGCAGTAC
MH_delAB_FOR	GTACTGCAGCACCTCGGCCTGGACGGTGG
MH_delAB_REV	GCGCGGACCCGCCTGCTC
MH_delbHP_FOR	GGCGGGTCCGCGCGTCCACCCGTTGTGGCTC
MH_delbHP_REV	GAATCCGGTGGTGGTGGTACGCTCGACACCGACCTG
Long_FOR	ACCACCACCACCGGATTCCAGGACATCGGCG
Long_REV	GGAGGCGGTGGAGGTGGTCTGCGCACCGCCAC
Medium_FOR	ACCACCTCCACCGCCTCCCTGCTCCTTGAGGTACTGGTTG
Medium_REV	AAGGGTGGCGGAGGTACCGTCCAGGCCTACGTG
	GGTACCTCCGCCACCCTTGAGGTACTGGTTGAACCGGTAC
**CPEC primers**	
pFLAG_FOR2	CGAGGCATGCGAAGGAGATATACATATG
pFLAG_REV2	CATATGTATATCTCCTTCGCATGCCTCG
**pINIT PCR primers**	
Rv1410c_for	atatGCTCTTCtAGTCGAGCAGGACGTCGAGTCGCG
Rv1410c_rev	tataGCTCTTCaTGCGAGCGGCTCCACTTGGGGCGCC

Supplementary Note 1: Rationale for mutant design

The tested mutations were always introduced to both Rv1410 and MHAS2168 to assure the relevance of the phenotype. The mutation sites were chosen according to our MHAS2168^{OUT} crystal structure or the MHAS2168^{IN} homology model, but the mutation locations were checked on Rv1410 structure predictions by ColabFold. Also, conservation in mycobacterial homologues in general (Supplementary Fig. 5) was taken into account. Production of each mutant was tested by Western blotting (Supplementary Fig. 4a,b), to ensure that the phenotypes are not due to insufficient protein production, aggregation, or degradation of the mutant transporters. What is more, we purified selected Rv1410 mutants displaying negative phenotypes in our vancomycin sensitivity assays from *E. coli* membranes to demonstrate that the phenotypes do not arise from aberrant folding or their incapability to insert into membranes (Supplementary Fig. 4c,d).

Unique ion lock mutations

The only acidic residue that could be protonated/deprotonated during transport cycle in the central cavity is E147$_{Mtb}$/E157$_{MH}$. This glutamate is fully conserved in 17 Rv1410 homologue proteins (Supplementary Fig. 5) and forms a salt bridge with a fully conserved arginine (R417$_{Mtb}$/R426$_{MH}$). This ion lock seems to be unique, as it is not commonly found in other MFS transporters (Supplementary Fig. 6). To test whether the protonation/deprotonation of the glutamate is important for transport, we introduced mutations E147Q$_{Mtb}$/E157Q$_{MH}$ to Rv1410 and MHAS2168, correspondingly. The glutamine cannot be deprotonated. In addition, we introduced the R417A$_{Mtb}$/R426A$_{MH}$ mutations to Rv1410 and MHAS2168, correspondingly, to investigate whether the formation of the ion lock is important for the transporter's activity.

D22/D35

In a paper by Farrow and Rubin[1], the D22 residue of Rv1410 was investigated as it was speculated to belong to a conserved motif D1. It was discovered that mutation D22A was as sensitive to ethidium as Rv1410 deletion mutant while D22E which also harbours a carboxylate group retained some of ethidium resistance, although not at wild-type level. We reasoned that if the importance of D22 lies in coupling proton translocation to substrate transport, D22N mutation should inactivate Rv1410. Therefore, we introduced the D22N$_{Mtb}$ and D35N$_{MH}$ mutations to Rv1410 and MHAS2168, correspondingly. This aspartate was fully conserved in 17 Rv1410 homologue proteins (Supplementary Fig. 5).

β-hairpin truncation

To assess whether the periplasmic β-hairpin found between TM9 and TM10 has any impact on the transporter's function, we decided to truncate the β-hairpin. To do that, we removed the two β-sheets forming the hairpin and residues connecting them, replacing them with a linker formed of four glycine residues. Therefore, Δβ-HP$_{MH}$ is a MHAS2168ΔR373-P382::GGGG mutant and Δβ-HP$_{Mtb}$ is a Rv1410ΔR363-P373::GGGG mutant.

Truncation of linker helices

To assess whether the linker helices TMA and TMB have any impact on the transporter's function, we decided to truncate these linker helices to turn Rv1410/MHAS2168 into a classical 12-helix MFS transporter. In this case, the lateral opening between TM2-TM11 in outward-facing conformation is not blocked by linker helices anymore. To achieve that, we deleted the linker helices TMA and TMB and the periplasmic loop connecting them. Since we deemed the remaining cytoplasmic loops to be long enough to connect N- and C-domains, we did not add any extra linker. Therefore, ΔAB_{MH} is a MHAS2168ΔV213-F265 mutant and ΔAB_{Mtb} is a Rv1410ΔL203-F255 mutant.

Mutations in lateral clefts

To investigate whether lateral openings between N- and C-domain could serve as the entry or exit points for TAGs, we adopted a mutation strategy similar to one used in assessment of MFSD2A[2]. However, we decided to introduce mutations to each of the four lateral clefts, forming on opposite sides of the transporter in both outward-facing and inward-facing conformations, assessed in our MHAS2168^{OUT} crystal structure and MHAS2168^{IN} homology model. We selected residues on TM2 and TM5 in the middle of each cleft whose side chains (if existing) were faced towards TM11 or TM8, correspondingly. We assumed that if these hydrophobic residues were mutated into glutamates or aspartates, the charged/polar side chains might prevent TAG diffusion through the lateral clefts. To choose whether a glutamate could be introduced into a given location, steric hindrances in each mutant were assessed *in silico* in both MHAS2168^{OUT} and MHAS2168^{IN} conformations. If the side-chain of glutamate seemed to encounter steric hindrances in either of the two conformations, an aspartate was introduced to the loci instead. Summary of the mutants in tabular form:

Lateral cleft	Mutation in Rv1410	Mutation in MHAS2168	Helices lining the lateral cleft	The lateral cleft is open in
**TM5-TM8^{OUT}**	L155E_{Mtb}	I165E_{MH}	TM5-TM8	Outward-facing state
**TM5-TM8^{IN}**	G140D_{Mtb}	G150D_{MH}	TM5-TM8	Inward-facing state
**TM2-TM11^{OUT}**	L422E_{Mtb}	L431E_{MH}	TM2-TM11	Outward-facing state
**TM2-TM11^{IN}**	A411D_{Mtb}	A420D_{MH}	TM2-TM11	Inward-facing state

Mutations in central cavity

The aim of these mutations was to introduce charge (and bulk) to the hydrophobic wall of the central cavity, in the hope that it might interfere with TAG transport if the molecule resides in the central cavity during its transport. L289$_{Mtb}$/L299$_{MH}$ was chosen as the mutation site because i) its side chain is located in the middle of the hydrophobic central cavity wall in the MHAS2168^{OUT} C-domain, ii) it is fully conserved in 17 Rv1410 homologue proteins (Supplementary Fig. 5), iii) the MHAS2168^{IN} homology model could accommodate a bulky residue in that position. Therefore, L289R_{Mtb}/L299R_{MH} mutations were introduced to Rv1410 and MHAS2168, correspondingly, to introduce charge and bulk into the cavity. As a control,

L289D$_{Mtb}$/L299D$_{MH}$ mutations were introduced to Rv1410 and MHAS2168, correspondingly, to introduce polar residues with similar size as the original leucine in that position.

Truncations of periplasmic helix extensions

In our previous work[3], we detected a unique "periplasmic loop" between TM11 and TM12 and investigated its truncation mutants which exhibited gradual loss of functionality, the more residues were removed (Long loop = Truncation 1[Δ10aa]; Medium loop = Truncation 2[Δ18aa]; Short loop [Δ26aa]). Our structure showed the presence of TM11 and TM12 helix extensions, instead of a periplasmic loop. When the structures of Rv1410 truncation mutants were predicted by ColabFold platform[4], surprisingly, the truncation mutants exhibited equal lengths of TM11 and TM12, even if TM12 N-terminal residues had to be conscripted to make up lost length of TM11 C-terminus or vice versa. We decided to design truncation mutants in MHAS2168 to confirm that loss of helix length incurs inactivity of the transporter. However, instead of deleting the corresponding residues of Rv1410 truncation mutants in MHAS2168, we decided to mimic the tertiary structure of these truncations by deleting residues from both TM11, TM12 and the loop connecting them, and introducing a glycine linker (of similar length to the original truncation mutants in Rv1410) between the remaining helices to ensure that the original helices are not disturbed. Therefore, Truncation 1$_{MH}$ is a MHAS2168ΔL453-R474::GGGGGG mutant and Truncation 2$_{MH}$ is MHAS2168ΔE451-A478::GGGG mutant.

TM12 mutations

Analysis of periplasmic helix extensions uncovered some conserved features that were common in all the Rv1410 mycobacterial homologues investigated by MSA (Supplementary Fig. 5) and ColabFold structure predictions (Supplementary Fig. 2). The residues on the tip of the TM12 extension (1st α-turn) directed towards the cavity are hydrophobic and aromatic residues are commonly found on TM12 above the cavity (whether on the 4th or 5th α-turn or both). Therefore, we decided to mutate these features to assess whether they might play a role in TAG transport. To judge the necessity for aromatic residues to TAG transport, we introduced alanine mutations Y464A$_{Mtb}$, F468A$_{Mtb}$, T479A$_{MH}$, Y483A$_{MH}$ to the sites of aromatic residues above cavity. To assess whether charged and bulkier side-chains at these locations might interfere with TAG transport, we introduced glutamate mutations Y464E$_{Mtb}$, F468E$_{Mtb}$, T479E$_{MH}$, Y483E$_{MH}$ to the aromatic residues above cavity. To investigate whether the hydrophobic tip of TM12 has a role in TAG transport, we introduced mutations with either positive (L453K$_{Mtb}$, M468K$_{MH}$) or negative charge (L453D$_{Mtb}$, M468D$_{MH}$) to this locus.

Supplementary Note 2: Description of MD simulations

Lipid interactions in MHAS2168^{OUT} and MHAS2168^{IN}

Coarse-grained molecular dynamics (MD) simulations of MHAS2168 were performed in both outward-open (MHAS2168^{OUT}) and inward-open conformations (MHAS2168^{IN}) embedded in a phospholipid bilayer doped with TAGs, mimicking the mycobacterial plasma membrane (see Methods; Supplementary Table 3; Fig. 3a). The MFS fold and the sequence similarity shared with other transporters from the same family allowed the generation of a structural model of MHAS2168 in the inward-facing conformation, using PepT$_{So2}$ as template (see Methods).

The embedment of the TAGs in the hydrophobic core of the membrane (Fig. 3b) enables them to probe a range of different positions along the transporter TM helices (Fig. 3c). TAGs visit all lateral openings of the transporter with a preference for TM8 and TM10 (Supplementary Fig. 7). Protein-lipid contact analyses show that TAGs are more strongly interacting with MHAS2168^{IN} (Supplementary Fig. 7; Supplementary Table 4), with a particular involvement of the TM4 N-terminus, TM7 N-terminus, TM12 C-terminus and the linker helices TMA and TMB.

Concerning the interactions of the transporter with the phospholipids, it is found that MHAS2168 strongly interacts with CDL and POPI lipids. Small differences are observed between the MHAS2168^{OUT} and MHAS2168^{IN} conformations (Supplementary Fig. 7; Supplementary Table 4). Different phospholipid interactions were found especially for the connecting loop between TM10 and TM11 (residues 409-415) and for the TM11 C-term (Supplementary Fig. 7), where MHAS2168^{IN} was found to interact more with POPI than MHAS2168^{OUT}.

TAGs in MHAS2168^{OUT} and MHAS2168^{IN} central cavities

We further investigated the central cavity and its interactions with TAG during the MD simulations of both conformations. The outward-facing conformation cavity volume was estimated to be ~2500 Å^3, while the inward-facing homology model displays a slightly larger central cavity and a broader distribution, centred around 2800 Å^3 (Supplementary Fig. 9).

Furthermore, we recorded the entrance events of the different lipid species into the central cavity of MHAS2168 during MD simulations (Supplementary Fig. 8). In our analysis, an entrance event is recorded when the phosphate bead of a phospholipid or the TAG backbone bead overlap with the central cavity volume shown in Supplementary Fig. 9. Apart from a few snorkelling events, in which a TAG molecule probes the periphery of the cavity but does not fully enter, the MHAS2168^{OUT} cavity is little accessed by phospholipids and TAGs. Visual inspection of the trajectories confirmed that no entrance events occurred in the simulations of the outward-facing conformation, neither for TAG nor for the phospholipids. Conversely, for the MHAS2168^{IN} system, multiple entry and exit events were observed for POPG, POPE and especially POPI (Supplementary Fig. 8). Phospholipid entrance and exit events occur via both lateral openings (i.e., TM5-TM8^{IN} and TM2-TM11^{IN}). However, no TAG molecule entered the

cavity in the MD simulations, likely because of the much lower TAG concentration compared to the competing phospholipid species. Spontaneous TAG binding was also not observed in additional control CG-MD simulations with a higher concentration of TAG in the bilayer (7%, corresponding to 14 TAG molecules, see Supplementary Table 3), likely due to the aggregation of the TAGs in the membrane midplane.

To probe whether the central cavity can accommodate TAG, we ran five additional MD simulations for MHAS2168^{IN}, where we inserted one TAG molecule into the main cavity from the beginning of the simulations (the MHAS2168$^{IN\text{-}TAG}$ system, see Supplementary Table 3, Supplementary Fig. 11). In all the MHAS2168$^{IN\text{-}TAG}$ simulated repeats the TAG initially positioned inside the main cavity escaped through the TM5-TM8^{IN} lateral cleft. In particular, the majority of the contacts involved helices TM5 (N-terminal), TM8 (C-terminal), TM10 (C-terminal) and TM11 (N-terminal) (see Supplementary Table 4). The TAG residence times within the central cavity in these 5 simulations are 20, 15, 18, 8 and 74 μs, respectively, enabling us to collect a total of 135 μs of simulation time that allow one to map the TAG interactions within the cavity (Supplementary Fig. 11). Especially the TM5 N-terminus and the TM8 C-terminus, where the non-proteinaceous density was found in the cryo-EM structure (Fig. 1a), preferably interact with TAG in the simulations (Supplementary Fig. 11).

In order to expand our investigation and to support our approach of positioning one TAG molecule within the central cavity, we carried out a molecular docking study on both the outward-facing and the inward-facing conformations. The docking procedure is reported in the Method section. Briefly, AutoDock-Vina[5,6] was used, with the transporter (i.e., the receptor) considered rigid. One molecule of TAG (i.e., the ligand) was docked, which was considered to be flexible in AutoDock-Vina. For the outward-facing conformation, our results show a consistent preferential hotspot where all the poses were found nearby helices 7, 11 and facing helices A and B (Supplementary Fig. 10a). However, no TAG was docked inside the central cavity. For the inward-facing conformation, three distinct hotspots where found: two towards the periplasmic leaflet and on both sides of the transporter, and one within the central cavity (see Supplementary Fig. 10c), thereby confirming that our inward-facing structural model can accommodate one molecule of TAG within the central main cavity. Consistent with the outward-facing conformer, TAG poses were found nearby helix 11 and facing helices A and B (Supplementary Fig. 10c), suggesting that this could be a potential "*reservoir*" where TAGs might accumulate in between transport cycles. In addition, the docking analysis reveals hotspots (Supplementary Fig. 10, dotted circles) in close proximity to residues identified in contact with TAG during the MD simulations.

To extend our analysis, we then performed a further cycle of molecular docking accounting for flexibility not only of the TAG molecule, but also of residues Glu157 and Arg426, which are located within the main cavity and hypothesized to be a candidate locus for H^+ translocation. All the rest of the protein was considered rigid. For the inward-facing conformation, we found the same hotspots (Supplementary Fig. 10d). However, for the

outward-facing conformation, in addition to the hotspot nearby helices 7, 11 and TMA-TMB, one TAG molecule was found within the main central cavity (Supplementary Fig. 10b). Overall, the docking results strengthen our initial guess/assumption that the MHAS2168 main central cavity can accommodate one TAG molecule.

TAG transfer from MHAS2168^{OUT} to LprG

In addition, we studied TAG interactions with the central cavity of MHAS2168^{OUT} and its transfer process from MHAS2168 to LprG (MHAS2168^{OUT}-LprG system, Supplementary Table 3). To achieve that, a complex of MHAS2168^{OUT} and LprG was built, utilizing the ColabFold[4] platform. In these simulations, TAG moved between the hydrophobic cavities of MHAS2168^{OUT} and LprG (see description in main text; Fig. 6; Supplementary Fig. 13; Supplementary Video 1).

Initially, the TAG was inserted into the transporter with 2 hydrophobic tails pointing upwards (towards LprG) and 1 tail downwards (embedded into the main cavity of MHAS2168, Fig. 6). To probe whether this particular initial configuration could bias the movement of TAG towards LprG, an additional control simulation was carried out in which the TAG molecule was docked with only 1 tail pointing upwards and 2 tails downwards (Supplementary Fig. 14). In this control simulation, the TAG initially remained in its 2-tails-down configuration for around 80 μs, but then spontaneously transitioned to a 2-tails-up configuration until the end of the simulation run at 100 μs (Supplementary Fig. 14). When this control simulation was extended (Supplementary Fig. 14, red line) or used as a starting point for additional 5 repeat simulations with different starting velocities (Supplementary Fig. 14, lines from 1 to 5), the TAG was found to be loaded into LprG, akin to the independent simulations having a 2-tails-up configuration at the onset of the simulation (Fig. 6). We thus conclude that the initial configuration of the TAG does not determine the outcome of the simulations, and that at least 2 tails of the TAG need to be first oriented towards LprG for the TAG molecule to be spontaneously transferred into the LprG hydrophobic cavity.

In our initial set of CG-MD simulations, harmonic bonds (the "elastic network") were used not only within the transporter and LprG, but also some bonds between these two proteins. While the use of such an elastic network is by default required in CG-Martini simulations of proteins, the definition of inter-protein elastic bonds might be considered to be somewhat arbitrary, especially in light of the fact that this is done on the basis of the distances between pairs of backbone beads, which for the MHAS2168-LprG intermolecular contacts solely result from the AlphaFold prediction. Therefore, we carried out additional control simulations in which we completely removed all inter-molecular (that is, between MHAS2168 and LprG) elastic bonds (Supplementary Fig. 15). Reassuringly, also in these simulations, TAG transfer from MHAS2168 into LprG was observed, supporting the robustness of our findings (Supplementary Fig. 15).

It is noteworthy that the dissociation of LprG from MHAS2168 was never observed in any of the simulations (on the 0.3 ms time scale). However, this should not be interpreted as the

existence of high-affinity interaction between MHAS2168 and LprG, but rather that the complex represents a local minimum on the free energy landscape. The heterodimer might nevertheless be rather short-lived and might be transiently stabilized by the presence of TAGs within the interface of the two proteins, which makes it hard to experimentally validate this interaction. The experimental time scale, 0.1 ms (for the simulations with the inter-molecular elastic bonds, Fig. 6) and 30 μs (for the control simulations, Supplementary Fig. 15) still accounts for a "transient" protein-protein complex, so these findings do not directly contradict previously reported lack of strong physical interactions between the proteins[3,7] or our data from the operon shuffling experiments (Fig. 5d-h).

Supplementary References

1. Farrow, M. F. & Rubin, E. J. Function of a mycobacterial major facilitator superfamily pump requires a membrane-associated lipoprotein. *J. Bacteriol.* **190**, 1783–1791 (2008).
2. Wood, C. A. P. *et al.* Structure and mechanism of blood–brain-barrier lipid transporter MFSD2A. *Nature* **596**, 444–448 (2021).
3. Hohl, M. *et al.* Increased drug permeability of a stiffened mycobacterial outer membrane in cells lacking MFS transporter Rv1410 and lipoprotein LprG. *Mol. Microbiol.* **111**, 1263–1282 (2019).
4. Mirdita, M. *et al.* ColabFold: making protein folding accessible to all. *Nat. Methods* **19**, 679–682 (2022).
5. Trott, O. & Olson, A. J. AutoDock Vina: Improving the speed and accuracy of docking with a new scoring function, efficient optimization, and multithreading. *J. Comput. Chem.* **31**, 455–461 (2010).
6. Eberhardt, J., Santos-Martins, D., Tillack, A. F. & Forli, S. AutoDock Vina 1.2.0: New Docking Methods, Expanded Force Field, and Python Bindings. *J. Chem. Inf. Model.* **61**, 3891–3898 (2021).
7. Touchette, M. H. *et al.* A Screen for Protein − Protein Interactions in Live Mycobacteria Reveals a Functional Link between the Virulence-Associated Lipid Transporter LprG and the Mycolyltransferase Antigen 85A. *ACS Infect Dis* **3**, 336–348 (2017).

Supplementary Source Data

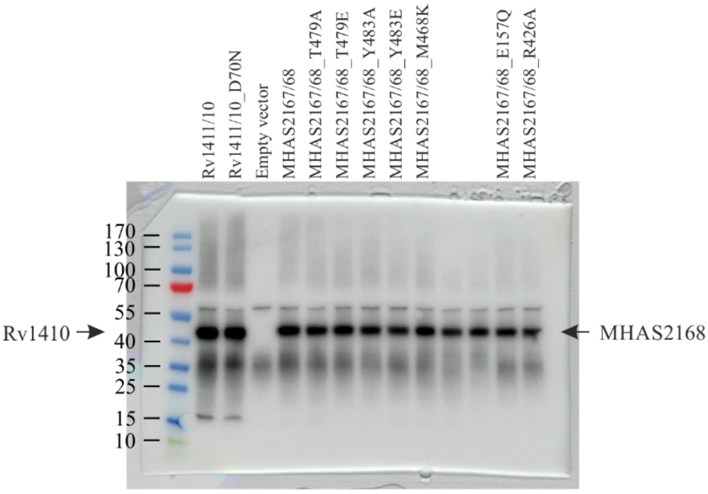

26.01.22

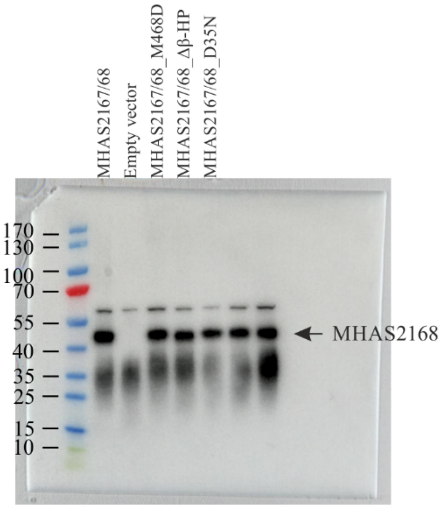

22.02.2022

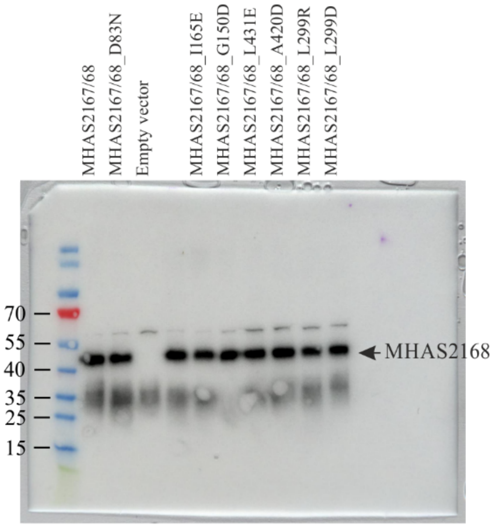

12.04.2022

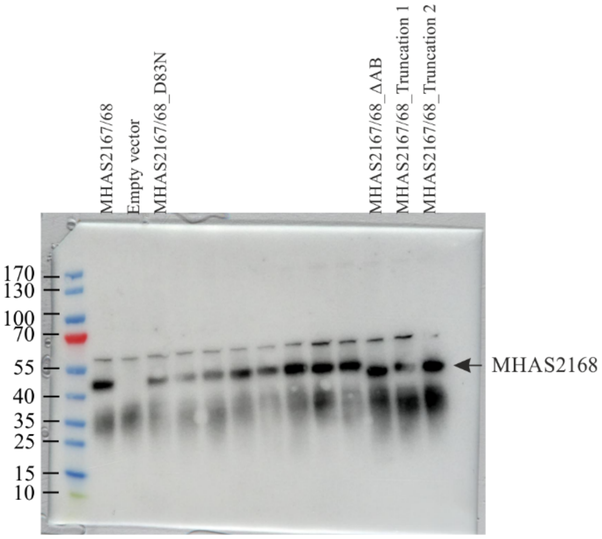

25.03.2022

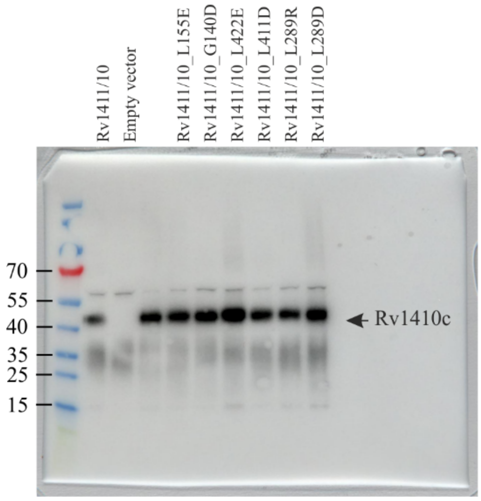

12.04.2022

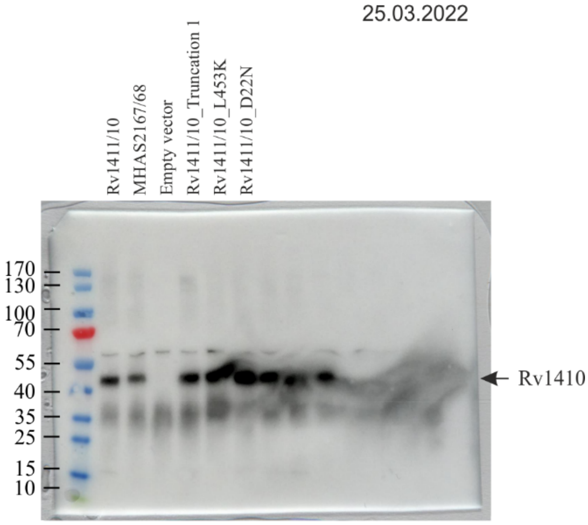

18.02.2022

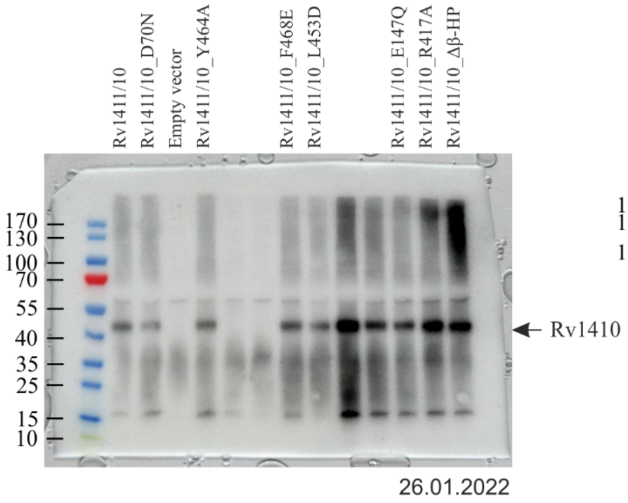
Rv1411/10
Rv1411/10_D70N
Empty vector
Rv1411/10_Y464A
Rv1411/10_F468E
Rv1411/10_L453D
Rv1411/10_E147Q
Rv1411/10_R417A
Rv1411/10_Δβ-HP
170
130
100
70
55
40
35
25
15
10
Rv1410
26.01.2022

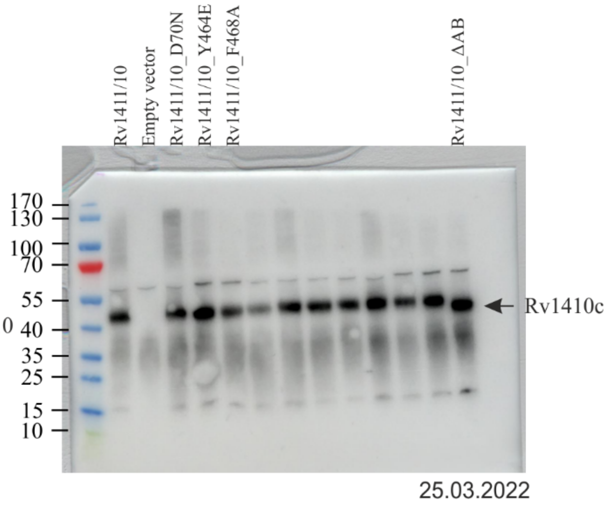
Rv1411/10
Empty vector
Rv1411/10_D70N
Rv1411/10_Y464E
Rv1411/10_F468A
Rv1411/10_ΔAB
170
130
100
70
55
40
35
25
15
10
Rv1410c
25.03.2022

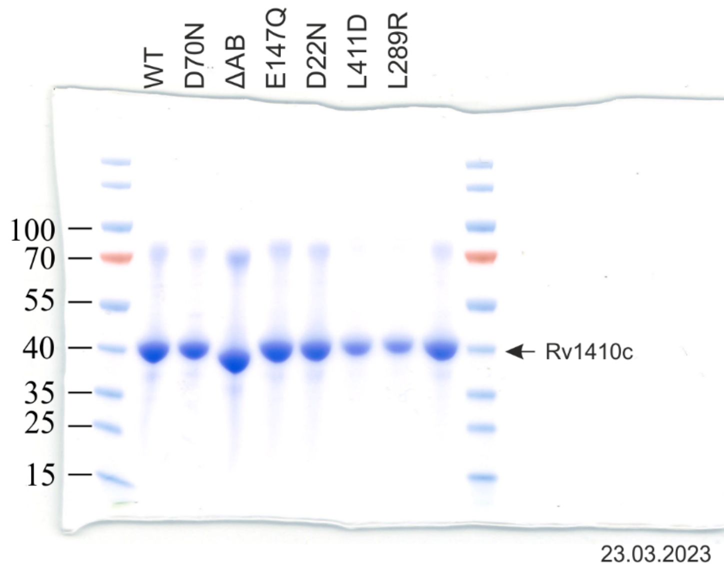
WT
D70N
ΔAB
E147Q
D22N
L411D
L289R
100
70
55
40
35
25
15
Rv1410c
23.03.2023
