# Supplementary material for: Structural basis for triacylglyceride extraction from mycobacterial inner membrane by MFS transporter Rv1410
Source: Nat Commun. 2023 Oct 13;14:6449. doi: 10.1038/s41467-023-42073-0 (PMC10576003; doi:10.1038/s41467-023-42073-0)
Supplement: Supplementary file 3 — Description of Additional Supplementary Files [file 41467_2023_42073_MOESM3_ESM.pdf]

File name: Supplementary Movie 1

Description: TAG transfer between MHAS2168 and LprG. The movie shows coarse-grained MD simulation of TAG transfer between MHAS2168 and LprG depicted on Figure 6a, replicate 1. MHAS2168 N-domain, light gray; MHAS2168 C-domain, dark gray; LprG, pale lilac. For clarity, the three TAG acyl tails have been colored differently (yellow, green, and purple).
