# Supplementary material for: Structural basis for triacylglyceride extraction from mycobacterial inner membrane by MFS transporter Rv1410
Source: Nat Commun. 2023 Oct 13;14:6449. doi: 10.1038/s41467-023-42073-0 (PMC10576003; doi:10.1038/s41467-023-42073-0)
Supplement: Supplementary file 5 — Reporting Summary [file 41467_2023_42073_MOESM5_ESM.pdf]

Corresponding author(s): Lars V. Schäfer and Markus A. Seeger

Last updated by author(s): Sep 28, 2023

Reporting Summary

Nature Portfolio wishes to improve the reproducibility of the work that we publish. This form provides structure for consistency and transparency in reporting. For further information on Nature Portfolio policies, see our Editorial Policies and the Editorial Policy Checklist.

Statistics

For all statistical analyses, confirm that the following items are present in the figure legend, table legend, main text, or Methods section.

n/a	Confirmed	
☐	☒	The exact sample size (*n*) for each experimental group/condition, given as a discrete number and unit of measurement
☐	☒	A statement on whether measurements were taken from distinct samples or whether the same sample was measured repeatedly
☒	☐	The statistical test(s) used AND whether they are one- or two-sided<br>*Only common tests should be described solely by name; describe more complex techniques in the Methods section.*
☒	☐	A description of all covariates tested
☒	☐	A description of any assumptions or corrections, such as tests of normality and adjustment for multiple comparisons
☐	☒	A full description of the statistical parameters including central tendency (e.g. means) or other basic estimates (e.g. regression coefficient) AND variation (e.g. standard deviation) or associated estimates of uncertainty (e.g. confidence intervals)
☒	☐	For null hypothesis testing, the test statistic (e.g. *F*, *t*, *r*) with confidence intervals, effect sizes, degrees of freedom and *P* value noted<br>*Give P values as exact values whenever suitable.*
☒	☐	For Bayesian analysis, information on the choice of priors and Markov chain Monte Carlo settings
☒	☐	For hierarchical and complex designs, identification of the appropriate level for tests and full reporting of outcomes
☒	☐	Estimates of effect sizes (e.g. Cohen's *d*, Pearson's *r*), indicating how they were calculated

Our web collection on statistics for biologists contains articles on many of the points above.

Software and code

Policy information about availability of computer code

Data collection	EPU-2.7 (Thermo Fisher Scientific)
Data analysis	cryoSPARC v3.2, coot-0.8.9.2-preEL, PHENIX 1.14-3260, ChimeraX 1.3, XDS BUILT=20190315 , ISOLDE 1.4, MODELLER 10.2, GROMACS version 2021.1, Martini 2.2 force field, CHARMM-GUI web server, insane tool 1.1-dev, trj_cavity_v2, VMD 1.9.4, Grace v5.1, CLUSTAL Omega, ColabFold 1.3.0, 3V: Voss Volume Voxelator, JalView 2.11.1.5, R 4.1.2, CLC Main Workbench 8.1.3

For manuscripts utilizing custom algorithms or software that are central to the research but not yet described in published literature, software must be made available to editors and reviewers. We strongly encourage code deposition in a community repository (e.g. GitHub). See the Nature Portfolio guidelines for submitting code & software for further information.

Data

Policy information about availability of data

All manuscripts must include a data availability statement. This statement should provide the following information, where applicable:

- Accession codes, unique identifiers, or web links for publicly available datasets
- A description of any restrictions on data availability
- For clinical datasets or third party data, please ensure that the statement adheres to our policy

The crystal structure of MHAS2168 in complex with Nb_H2 has been deposited in RCSB Protein Data Bank (PDB) with the accession code 8PNL. The cryo-EM map of MHAS2168 in complex with Mb_H2 has been deposited in Electron Microscopy Data Bank (EMDB) with the accession code EMD-17787.

The sequence of nanobody Nb_H2 has been made accessible through deposition of the PDB. The expression vector for the production of Nb_H2 is available from the authors upon request.

Human research participants

Policy information about studies involving human research participants and Sex and Gender in Research.

Reporting on sex and gender	NA
Population characteristics	NA
Recruitment	NA
Ethics oversight	NA

Note that full information on the approval of the study protocol must also be provided in the manuscript.

Field-specific reporting

Please select the one below that is the best fit for your research. If you are not sure, read the appropriate sections before making your selection.

☒ Life sciences ☐ Behavioural & social sciences ☐ Ecological, evolutionary & environmental sciences

For a reference copy of the document with all sections, see nature.com/documents/nr-reporting-summary-flat.pdf

Life sciences study design

All studies must disclose on these points even when the disclosure is negative.

Sample size	Sample size estimation was not relevant for this study, as it does not report on a statistical evaluation of effects between two or more groups.
Data exclusions	No data were excluded from the analysis.
Replication	Growth curves shown in Fig. 2c and d, Fig. 3d and e, Fig. 4e and f, Fig. 5b-h are representative data of at least 3 biological replicates and each data points corresponds to the mean of four technical replicates.<br>Data of all biological replicates of Rv1410/MHAS2168 mutants are shown in Figure S8.<br><br>Molecular dynamics analyses shown in Fig. 3a-c and Figure S7 are representative data of five independent production simulations conducted for 20 us.<br>Molecular dynamics analyses shown in Fig. 6 correspond to five independent production simulations conducted for 100 us. The corresponding TAG interactions with Rv1410-LprG of all five repeats are shown in Figure S9.<br>Molecular dynamics analyses shown in Fig. S14 correspond to a single simulation run for 100 us, followed by six independent extended simulation for at least another 50 us.<br><br>Western Blots shown in Figure S4 have been performed once. Each panel shows data obtained on the same day under identical experimental conditions. Each panel contains WT Rv1410 or MHAS2168 and empty vector (EV) control as positive and negative control, respectively.
Randomization	No randomization of sample was performed, because we did not draw any conclusions from comparing groups of transporter mutants.
Blinding	The authors were not blinded, because no subjective analyses were performed.

Reporting for specific materials, systems and methods

We require information from authors about some types of materials, experimental systems and methods used in many studies. Here, indicate whether each material, system or method listed is relevant to your study. If you are not sure if a list item applies to your research, read the appropriate section before selecting a response.

Materials & experimental systems

n/a	Involved in the study
☐	☒ Antibodies
☒	☐ Eukaryotic cell lines
☒	☐ Palaeontology and archaeology
☐	☒ Animals and other organisms
☒	☐ Clinical data
☒	☐ Dual use research of concern

Methods

n/a	Involved in the study
☒	☐ ChIP-seq
☒	☐ Flow cytometry
☒	☐ MRI-based neuroimaging

Antibodies

Antibodies used

Nanobodies Nb_H2 and Nb_F7 binding to MHAS2168 and Rv1410, respectively, were generated in alpacas as part of this study. To detect transporter mutants by Western blotting, we used α-FLAG antibody (Sigma, F3165) and α-mouse-HRP antibody (Sigma, A5278).

Validation

Binding of Nb_H2 to purified MHAS2168 was validated by ELISA, size exclusion chromatography and by structural analyses (cryo-EM and X-ray crystallography).
Binding of Nb_F7 to purified Rv1410 was validated by ELISA, size exclusion chromatography and by cryo-EM.

Animals and other research organisms

Policy information about studies involving animals; ARRIVE guidelines recommended for reporting animal research, and Sex and Gender in Research

Laboratory animals

One male, two year old alpaca (Vicugna pacos) called Vesuv was used for co-immunization with Rv1410 and MHAS2168.

Wild animals

No wild animals were used in this study.

Reporting on sex

Not relevant for the study, because no physiological conclusions for the alpaca was derived from animal experiment.

Field-collected samples

No field-collected samples were part of this study.

Ethics oversight

Immunizations of alpacas were approved by the Cantonal Veterinary Office in Zurich, Switzerland (animal experiment licence nr. 172/2014).

Note that full information on the approval of the study protocol must also be provided in the manuscript.
